# Development of a clinical polygenic risk score assay and reporting workflow

**DOI:** 10.1038/s41591-022-01767-6

**Published:** 2022-04-18

**Authors:** Limin Hao, Peter Kraft, Gabriel F. Berriz, Elizabeth D. Hynes, Christopher Koch, Prathik Korategere V Kumar, Shruti S. Parpattedar, Marcie Steeves, Wanfeng Yu, Ashley A. Antwi, Charles A. Brunette, Morgan Danowski, Manish K. Gala, Robert C. Green, Natalie E. Jones, Anna C. F. Lewis, Steven A. Lubitz, Pradeep Natarajan, Jason L. Vassy, Matthew S. Lebo

**Affiliations:** 1 Laboratory for Molecular Medicine, Mass General Brigham Personalized Medicine, Cambridge, MA USA; 2 Department of Epidemiology, Harvard T.H. Chan School of Public Health, Boston, MA USA; 3 Medical Genetics, Massachusetts General Hospital, Boston, MA USA; 4 Veterans Affairs Boston Healthcare System, Boston, MA USA; 5 Division of Gastroenterology, Massachusetts General Hospital, Boston, MA USA; 6 Harvard Medical School, Boston, MA USA; 7 Broad Institute of Harvard and the Massachusetts Institute of Technology, Cambridge, MA USA; 8 Department of Medicine, Brigham and Women’s Hospital, Boston, MA USA; 9 Precision Population Health, Ariadne Labs, Boston, MA USA; 10 E J Safra Center for Ethics, Harvard University, Cambridge, MA USA; 11 Cardiovascular Disease Initiative, Broad Institute of Harvard and the Massachusetts Institute of Technology, Cambridge, MA USA; 12 Cardiovascular Research Center, Massachusetts General Hospital, Boston, MA USA; 13 Demoulas Center for Cardiac Arrhythmias, Massachusetts General Hospital, Boston, MA USA; 14 Department of Pathology, Brigham and Women’s Hospital, Boston, MA USA

**Keywords:** Risk factors, Population genetics, Disease prevention

## Abstract

Implementation of polygenic risk scores (PRS) may improve disease prevention and management but poses several challenges: the construction of clinically valid assays, interpretation for individual patients, and the development of clinical workflows and resources to support their use in patient care. For the ongoing Veterans Affairs Genomic Medicine at Veterans Affairs (GenoVA) Study we developed a clinical genotype array-based assay for six published PRS. We used data from 36,423 Mass General Brigham Biobank participants and adjustment for population structure to replicate known PRS–disease associations and published PRS thresholds for a disease odds ratio (OR) of 2 (ranging from 1.75 (95% CI: 1.57–1.95) for type 2 diabetes to 2.38 (95% CI: 2.07–2.73) for breast cancer). After confirming the high performance and robustness of the pipeline for use as a clinical assay for individual patients, we analyzed the first 227 prospective samples from the GenoVA Study and found that the frequency of PRS corresponding to published OR > 2 ranged from 13/227 (5.7%) for colorectal cancer to 23/150 (15.3%) for prostate cancer. In addition to the PRS laboratory report, we developed physician- and patient-oriented informational materials to support decision-making about PRS results. Our work illustrates the generalizable development of a clinical PRS assay for multiple conditions and the technical, reporting and clinical workflow challenges for implementing PRS information in the clinic.

## Abstract (web summary)

The first report from the prospective GenoVA Study provides preliminary insights into the development of a polygenic risk score assay in a clinical setting and discusses the challenges of generating, interpreting and reporting results.

## Main

Genome-wide association studies (GWAS) have identified thousands of genomic variants significantly associated with a range of common complex human diseases^1,2^. Given that the risk conferred by an individual common variant is usually insignificantly small, investigators have aggregated risk alleles across the genome into genetic risk scores to provide a single measure of genetic association for a given trait due to known common variant effects. Although the earliest genetic scores consisted only of variants meeting genome-wide significance^3–5^, recent computational and methodological advances have leveraged the summary statistics of all available variants from increasingly larger GWAS to calculate polygenic risk scores (PRS)^6–9^. For some diseases, a PRS in the upper tail of the distribution approximates risks equivalent to those conferred by established clinical risk factors and by genetic variants associated with monogenic disease^7,10^. Although PRS are typically derived from weights from cross-sectional GWAS of prevalent disease cases and controls, further work has demonstrated their potential to estimate the risk of incident disease^11–14^.

**Fig. 1:**
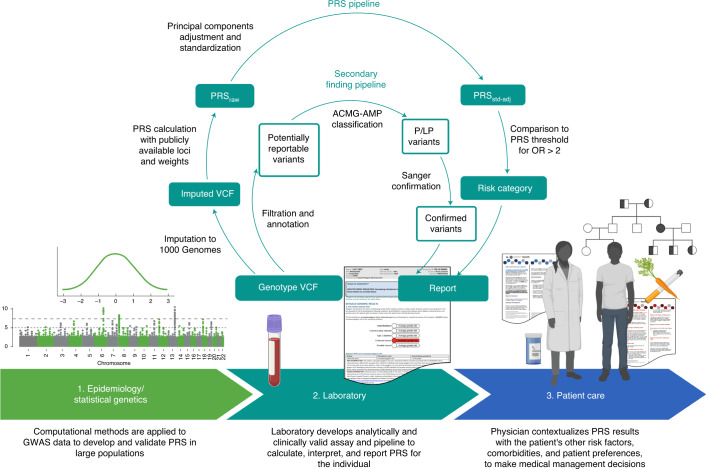
Translation of PRS from discovery to the clinic, including a clinical PRS laboratory pipeline for prospectively collected samples. In phase 1, PRS are developed, validated and compared to optimize performance in large populations. In phase 2, a clinical laboratory chooses publicly available PRS to implement and develop an analytically and clinically valid assay. For the GenoVA Study, genotype array data are imputed against 1000 Genomes Project data and used to calculate published PRS (PRS_raw_). PRS_raw_ is adjusted for population structure and standardized as described in the text (PRS_std-adj_). High-risk status for each disease is defined as PRS values above published thresholds for OR > 2. A parallel pipeline annotates and filters variants for potentially actionable pathogenic (P) and likely pathogenic (LP) variants in the ACMG SF v2.0 secondary finding gene list. Variants are manually classified according to American College of Medical Genetics and Genomics–Association for Molecular Pathology (ACMG-AMP) criteria by qualified laboratorians and confirmed using Sanger sequencing. Results from both components of the pipeline are included on the laboratory report. In phase 3 the treating physician uses the whole patient context to interpret the significance of the PRS for the patient’s health and healthcare management. Both the physician and patient will probably need educational and consultative support to make medical decisions based on PRS results.

Suitable clinical implementation of PRS is now an area of active research across many disease areas^15–17^. The translation of PRS from discovery to the clinic can be conceptualized as having at least three necessary phases (Fig. 1): the first phase relates to epidemiology and statistical genetics, in which PRS are developed and validated in large cohorts and improved with advances in statistical methods; the second phase involves the laboratory, in which laboratory geneticists must develop an analytically and clinically valid pipeline for calculating, interpreting and reporting PRS results for an individual patient; and the third phase involves patient care, in which a treating physician makes medical decisions after putting a patient’s PRS results into the larger clinical context, which involves non-genetic risk factors, comorbidities and patient preferences. The first phase has seen significant methodological advances^18^ but challenges for the second and third phases remain.

A key assumption underlying the laboratory phase is that a laboratory can develop and implement a valid clinical assay and interpretation pipeline to report PRS results for an individual patient. The development of a clinical assay from a published PRS is not trivial, and significant barriers to the process persist. First, uncertainty exists about whether commonly used, cost-effective genotyping arrays and clinical imputation pipelines can calculate a PRS for an individual with the analytic validity expected of a clinical assay, as opposed to one that is adequate for research. Second, laboratories must implement methods to account for the reduced validity of most PRS in patients of non-European and admixed ancestry^19,20^. This limitation applies both to the calculation of the PRS itself for an individual patient and to its clinical interpretation, given that published effect sizes are from populations of primarily European ancestry^19^. Third, laboratories must make several decisions about the content and format of a clinical PRS report, including decisions about where the laboratory’s role as an interpretative service ends and where the role of the treating physician in patient care begins. In the patient care phase, there remain unanswered questions about the information and support that physicians need when contextualizing the PRS results of an individual patient to make clinical decisions, and how those decisions affect patient outcomes.

In the Genomic Medicine at Veterans Affairs (GenoVA) Study (ClinicalTrials.gov identifier: NCT04331535) we have developed processes to advance the laboratory and patient care phases of the clinical translation of PRS. The GenoVA Study is a clinical trial in which patients and their primary care physicians receive a clinical PRS laboratory report on five diseases commonly screened for and initially managed in primary care: coronary artery disease (CAD), type 2 diabetes mellitus (T2D), atrial fibrillation (AFib), colorectal cancer (CRCa), and either prostate cancer (PrCa) in male patients or breast cancer (BrCa) in female patients. Because the objectives of the GenoVA Study are to observe how PRS impact existing disease screening and diagnosis paradigms and enable increased detection of undiagnosed prevalent or newly incident disease, eligible patients have no known diagnoses of the target diseases and are aged 50–70 years, an age range during which much guideline-recommended screening and diagnosis of new disease occurs. Here, we describe the processes created in the GenoVA Study to develop and validate a genotype array-based clinical assay and report for six PRS and to support their effective translation into clinical care by the treating physicians.

## Results

### Replication of published PRS

#### Sample characteristics

**Fig. 2:**
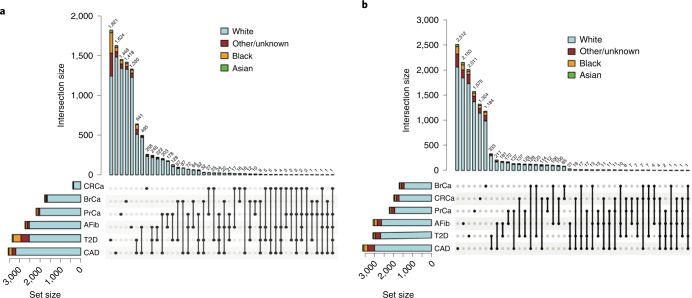
Frequency of disease and high-risk PRS results by race in the MGBB. **a**, UpSet plot of total cases of each of six phenotypes in 36,423 biobank participants and the counts of participants with one or more diseases, by reported race. **b**, UpSet plot of total counts of high-risk PRS results (population structure-adjusted PRS corresponding to OR > 2) for each of six diseases and the counts of participants with one or more high-risk PRS_std-adj_ results, by reported race.

To demonstrate the accuracy of a prospective PRS pipeline, we first wanted to ensure that we could implement published PRS effectively. We used data from 36,423 Mass General Brigham Biobank (MGBB) participants to replicate the performance of PRS for the six target diseases (Supplementary Table 1). The mean (s.d.) age of MGBB participants was 58.8 (17.1) years (range, 9–106 years), 19,719 (54.1%) were female, and 5,706 (15.7%) were of reported race other than white (white, *n* = 30,716 (84.3%); Black, *n* = 1,807 (5.0%); Asian, *n* = 786 (2.2%); and other or unknown race, *n* = 3,113 (8.5%) as determined from electronic health record data). Case counts ranged from 392 for CRCa to 3,554 for CAD. Figure 2a shows the counts of participants with one or multiple target diseases. The most common disease co-occurrences were the combinations of CAD and T2D (*n* = 641) and CAD and AFib (*n* = 495).

#### Unadjusted and adjusted PRS distributions

**Fig. 3:**
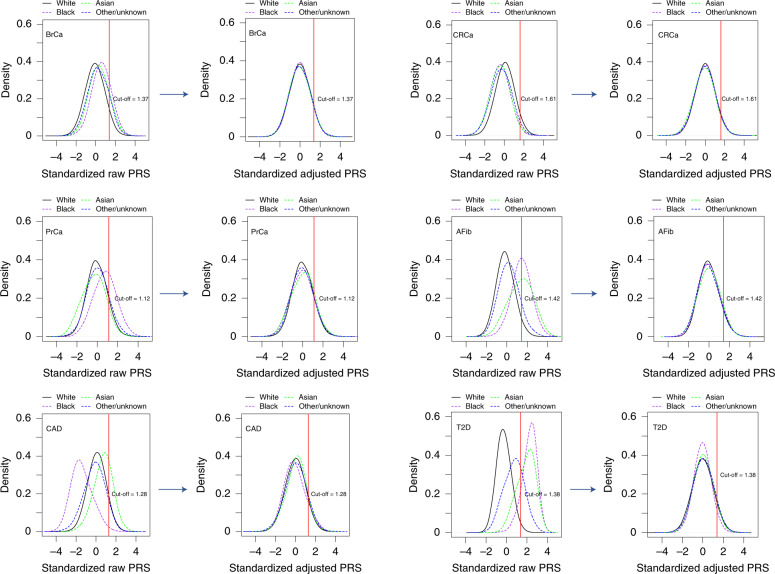
PRS distributions by reported race before and after adjustment for population structure. Plots to the left of each arrow show the distributions of unadjusted published PRS (PRS_std-raw_) by race for each of six diseases in up to 36,423 MGBB participants. Plots to the right of each arrow show these distributions after adjustment for population structure (PRS_std-adj_), as described in the text. The red vertical line indicates the standardized PRS threshold corresponding to OR > 2 for each disease, based on the OR per standard deviation from the original publication.

**Extended Data Fig. 1:**
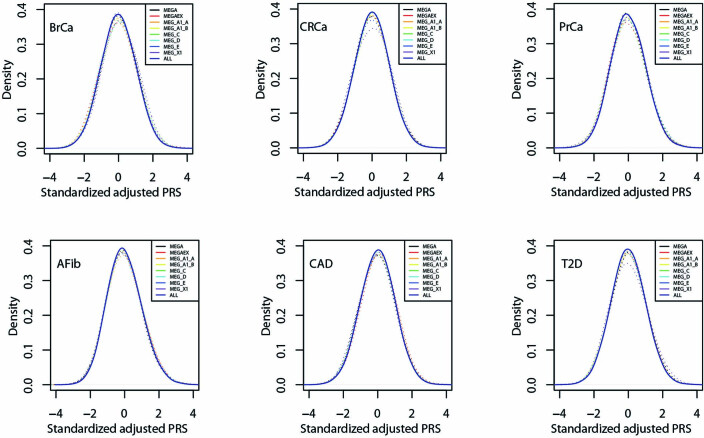
Distribution of standardized, adjusted PRS by release batch for six diseases in MGBB. Standardized, adjusted PRS (PRS_std-adj_) plotted by eight batches of three versions of Illumina genotyping arrays (MEG, MEGA, MEGAEX) used to analyze data from up to 36,423 MGBB participants. Abbreviations: AFib, atrial fibrillation; BrCa, breast cancer; CAD, coronary artery disease; CRCa, colorectal cancer; MEG, Multi-Ethnic Global; MEGA, Multi-Ethnic Genotyping Array; MEGAEX, Expanded Multi-Ethnic Genotyping Array; MGBB, Mass General Brigham Biobank; PrCa, prostate cancer; PRS, polygenic risk score; T2D, type 2 diabetes.

**Extended Data Fig. 2:**
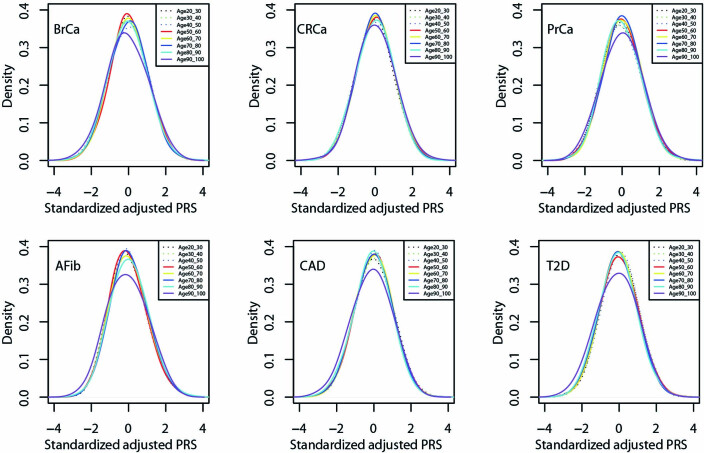
Distribution of standardized, adjusted PRS by age for six diseases in MGBB. Standardized, adjusted PRS (PRS_std-adj_) plotted by decade of age among up to 36,423 MGBB participants. Abbreviations: AFib, atrial fibrillation; BrCa, breast cancer; CAD, coronary artery disease; CRCa, colorectal cancer; MGBB, Mass General Brigham Biobank; PrCa, prostate cancer; PRS, polygenic risk score; T2D, type 2 diabetes.

**Extended Data Fig. 3:**
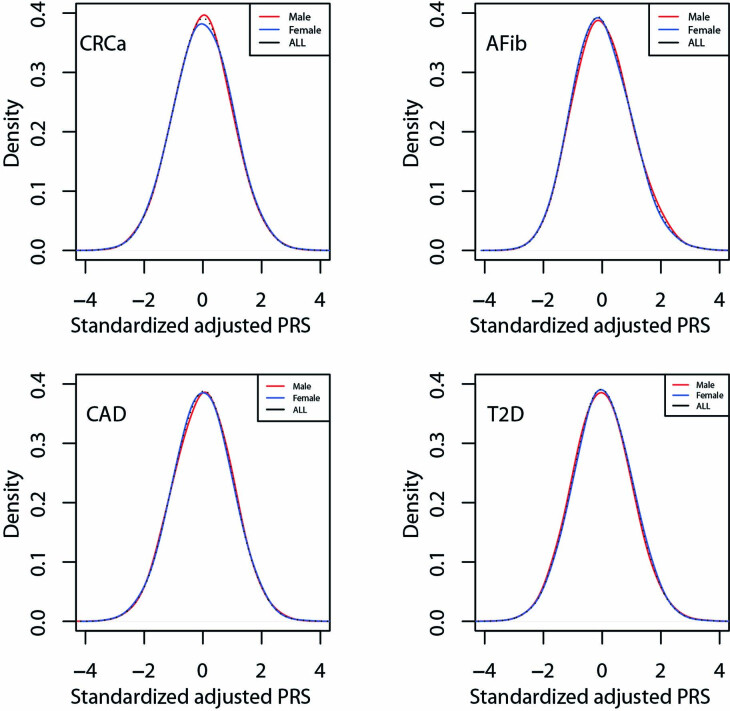
Distribution of adjusted PRS by sex for four diseases in MGBB. Standardized, adjusted PRS plotted by sex among 16,704 male and 19,719 female MGBB participants. Abbreviations: AFib, atrial fibrillation; BrCa, breast cancer; CAD, coronary artery disease; CRCa, colorectal cancer; MGBB, Mass General Brigham Biobank; PrCa, prostate cancer; PRS, polygenic risk score; T2D, type 2 diabetes.

We identified PRS from large GWAS for the six target diseases, for which the summary statistics (base files with alleles and weights) were publicly available from the Polygenic Score Catalog^21^ (AFib, CAD, T2D, BrCa) or the Cancer PRSWeb (CRCa, PrCa)^22^ as of 26 December 2019. Supplementary Table 2 lists the number of single-nucleotide polymorphisms (SNPs) in the base file for each of the six published PRS, ranging from 81 SNPs for CRCa^23^ to 6,917,436 for T2D^7^, and the subsets of these available as directly genotyped or imputed data from each of three arrays used for MGBB participants, demonstrating minimal loss of information compared with the original published PRS. As shown in Fig. 3, when using the published weights to calculate standardized PRS (PRS_std-raw_, see Methods) we observed marked variation in the distribution of each PRS by reported race in the MGBB, most notably in AFib, CAD, and T2D. For example, only 1.7% of white MGBB participants (516/30,716) but almost all of the Black MGBB participants (88.9%, 1,606/1,807) had PRS_std-raw_ above the published threshold associated with an odds ratio (OR) = 2 for T2D in the 2018 study by Khera et al.^7^ (Supplementary Tables 3 and 4). The use of residualized, population structure-adjusted, standardized PRS (PRS_std-adj_, see Methods) minimized this variation (Fig. 3), such that, for example, 8.6% of white MGBB participants (2,651/30,716) and 4.2% of Black MGBB participants (75/1,807) had a T2D PRS_std-adj_ above the published OR > 2 threshold. The distributions of PRS_std-adj_ were well aligned when examined by genotyping batch, decile of age, and sex (Extended Data Figs. 1–3).

#### Replication of PRS–disease association

**Fig. 4:**
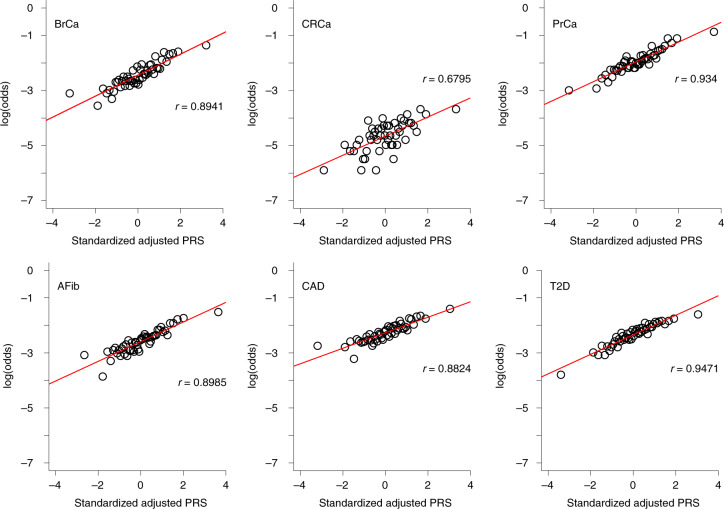
Correlation between adjusted PRS and odds of disease. The plots show log(odds) of each of six diseases versus quantile (*n* = 50) of standardized population structure-adjusted PRS (PRS_std-adj_) in up to 36,423 MGBB participants.

**Extended Data Fig. 4:**
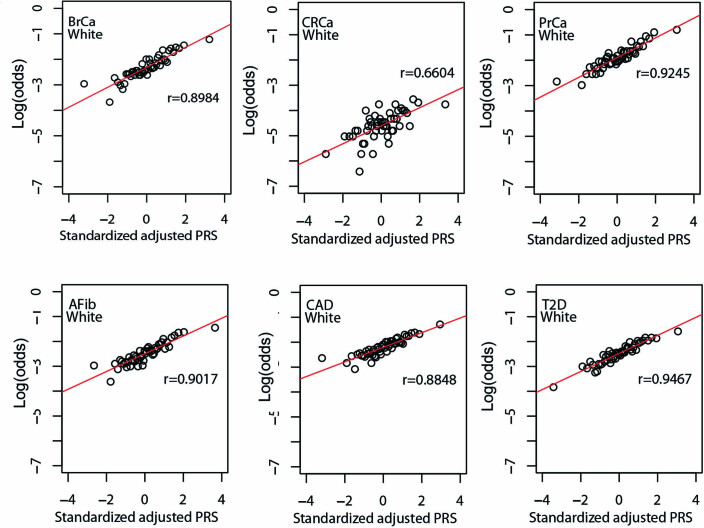
Correlation between standardized, adjusted PRS and odds of disease in reported white MGBB participants. Plots show log(odds) of each of six diseases versus quantile (n = 50) of standardized population structure-adjusted PRS (PRS_std-adj_) among up to 30,716 MGBB participants of reported white race. The correlation coefficient, r, is shown in each panel. Abbreviations: AFib, atrial fibrillation; BrCa, breast cancer; CAD, coronary artery disease; CRCa, colorectal cancer; MGBB, Mass General Brigham Biobank; OR, odds ratio; PrCa, prostate cancer; PRS, polygenic risk score; T2D, type 2 diabetes.

**Extended Data Fig. 5:**
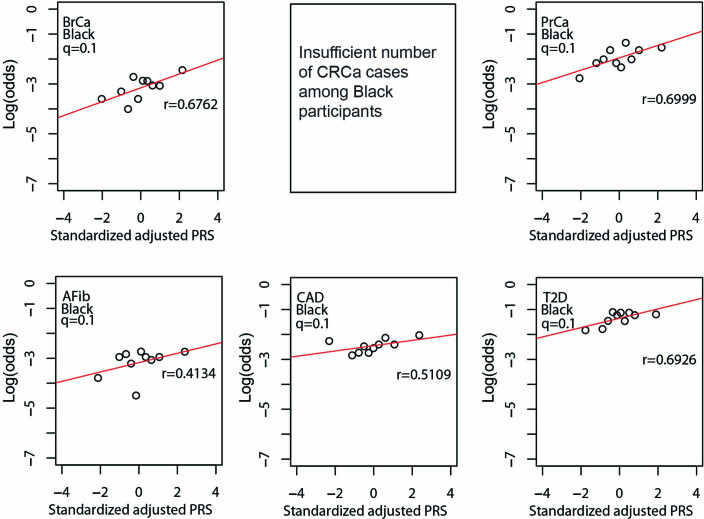
Correlation between standardized, adjusted PRS and odds of disease in reported Black MGBB participants. Plots show log(odds) of each of six diseases versus quantile (n = 10) of standardized population structure-adjusted PRS (PRS_std-adj_) among up to 1,807 MGBB participants of reported Black race. Results not reported for CRCa due to 0 CRCa cases in at least one quantile. The correlation coefficient, r, is shown in each panel. Abbreviations: AFib, atrial fibrillation; BrCa, breast cancer; CAD, coronary artery disease; CRCa, colorectal cancer; MGBB, Mass General Brigham Biobank; OR, odds ratio; PrCa, prostate cancer; PRS, polygenic risk score; T2D, type 2 diabetes.

**Extended Data Fig. 6:**
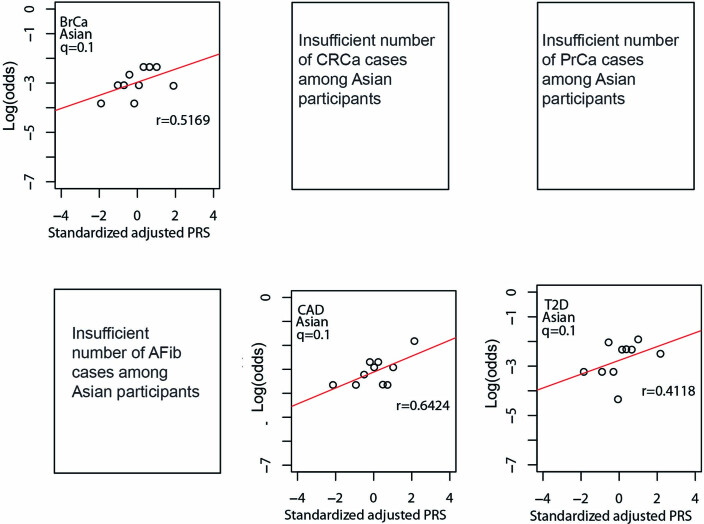
Correlation between standardized, adjusted PRS and odds of disease in reported Asian MGBB participants. Plots show log(odds) of each of six diseases versus quantile (n = 10) of standardized population structure-adjusted PRS (PRS_std-adj_) among up to 786 MGBB participants of reported Asian race. Results not reported for CRCa, PrCa, or AFib due to 0 cases in at least one quantile. The correlation coefficient, r, is shown in each panel. Abbreviations: AFib, atrial fibrillation; BrCa, breast cancer; CAD, coronary artery disease; CRCa, colorectal cancer; MGBB, Mass General Brigham Biobank; OR, odds ratio; PrCa, prostate cancer; PRS, polygenic risk score; T2D, type 2 diabetes.

**Extended Data Fig. 7:**
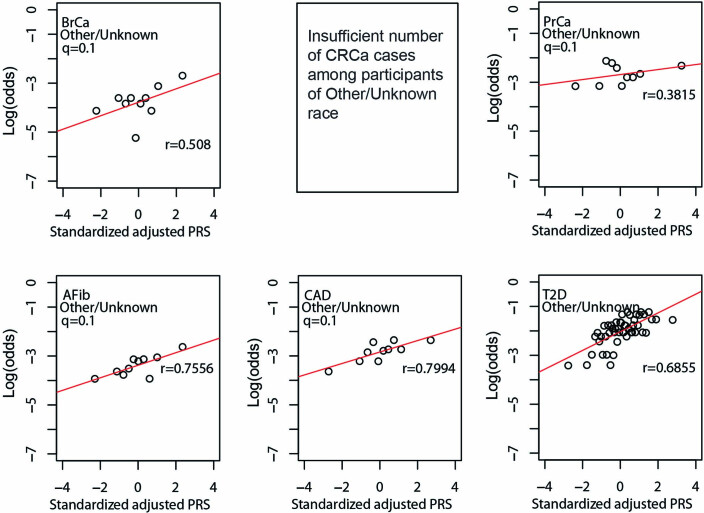
Correlation between standardized, adjusted PRS and odds of disease in MGBB participants of unknown or other reported race. Plots show log(odds) of each of six diseases versus quantile (n = 50 for T2D, n = 10 for all other disease) of standardized population structure-adjusted PRS (PRS_std-adj_) among up to 3,113 MGBB participants of unknown or other reported race. Results not reported for CRCa due to 0 cases in at least one quantile (n = 10). The correlation coefficient, r, is shown in each panel. Abbreviations: AFib, atrial fibrillation; BrCa, breast cancer; CAD, coronary artery disease; CRCa, colorectal cancer; MGBB, Mass General Brigham Biobank; OR, odds ratio; PrCa, prostate cancer; PRS, polygenic risk score; T2D, type 2 diabetes.

As shown in Fig. 4, quantile of PRS_std-adj_ was highly correlated with log(odds) of disease across the six phenotypes in the MGBB, with correlation coefficients ranging from 0.68 for CRCa to 0.95 for T2D. Extended Data Figs. 4–7 show the correlation of PRS_std-adj_ quantile and log(odds) of disease in the reported racial groups separately. Our analyses also replicated the published PRS thresholds corresponding to OR > 2. As shown in Table 1, at the published PRS_std-adj_ thresholds we observed OR ranging from 1.75 (95% CI: 1.57–1.95) for T2D to 2.38 (95% CI: 2.07–2.73) for BrCa in MGBB participants overall. Except for T2D, the 95% confidence interval of the replicated OR for all diseases either included or, in the case of BrCa and AFib, exceeded a point estimate of 2. Results were consistent in analyses restricted to white participants but were variable in other groups, largely because of the small number of disease cases in certain racial subgroups. In 22 of 24 analyses stratified by reported race, subjects with PRS_std-adj_ above the published OR > 2 thresholds had higher odds of disease than those below these thresholds. In the MGBB overall, the prevalence of a high-risk PRS_std-adj_ ranged from 5.4% for CRCa to 13.2% for PrCa (in men). Figure 2b illustrates the number of participants with PRS_std-adj_ above the published OR > 2 threshold for one or more of the target diseases. Of note, similar to the disease co-occurrences observed in MGBB participants, the most common co-occurrences of high-risk PRS_std-adj_ were the combinations of CAD and T2D (*n* = 333) and CAD and AFib (*n* = 211).

**Table 1:** Prevalence and disease associations of high-risk PRS for six diseases in MGBB overall and by reported race

Disease	High risk (%)^a^	OR overall	OR white	OR Black	OR Asian	OR Other/Unknown
		OR (95% CI)^b^ (*n*/*n*, *n*/*n*)^c^	OR (95% CI)^b^ (*n*/*n*, *n*/*n*)^c^	OR (95% CI)^b^ (*n*/*n*, *n*/*n*)^c^	OR (95% CI)^b^ (*n*/*n*, *n*/*n*)^c^	OR (95% CI)^b^ (*n*/*n*, *n*/*n*)^c^
BrCa	8.6	2.38 (2.07–2.73) (286/1,400, 1,427/16,606)	2.39 (2.07–2.76) (270/1,156, 1,318/13,495)	2.24 (0.97–5.15) (7/73, 43/1004)	0.51 (0.07–3.9) (1/33, 24/405)	2.35 (1.08–5.1) (8/138, 42/1,702)
CRCa	5.4	2.37 (1.74–3.24) (46/1,913, 346/34,117)	2.29 (1.65–3.19) (41/1,646, 312/28,717)	4.11 (1.17–14.48) (3/83, 15/1706)	0 (0–NaN) (0/35, 7/744)	3.30 (0.73–14.88) (2/149, 12/2,950)
PrCa	13.1	2.22 (1.98–2.48) (498/1,698, 1,693/12,813)	2.31 (2.05–2.59) (468/1,448, 1,544/11,017)	1.39 (0.74–2.59) (14/71, 74/521)	2.58 (0.5–13.28) (2/36, 6/279)	1.41 (0.78–2.58) (14/143, 69/996)
AFib	8.3	2.37 (2.12–2.64) (450/2,589, 2,282/31,101)	2.40 (2.14–2.69) (422/2,179, 2,101/26,014)	1.47 (0.72–3.01) (9/137, 71/1590)	2.00 (0.57–7.03) (3/62, 17/704)	2.28 (1.32–3.94) (16/211, 93/2,793)
CAD	9.8	1.86 (1.69–2.05) (562/3,018, 2,991/29,851)	1.91 (1.73–2.12) (503/2,459, 2,680/25,074)	1.41 (0.86–2.29) (21/177, 125/1484)	3.96 (1.79–8.76) (9/51, 31/695)	1.47 (0.97–2.22) (29/331, 155/2,598)
T2D	8.4	1.75 (1.57–1.95) (439/2,612, 2,924/30,447)	1.93 (1.71–2.17) (367/2,284, 2,159/25,906)	1.21 (0.7–2.09) (18/57, 358/1374)	1.07 (0.37–3.08) (4/49, 52/681)	1.58 (1.14–2.19) (50/222, 355/2,486)

High-risk PRS, defined here as a standardized, adjusted PRS (PRS_std-adj_) associated with OR > 2 for disease in the original publication. ^a^Proportion of MGBB participants exceeding the literature-derived OR > 2 threshold for each disease. ^b^Observed OR (95% CI) in up to 36,423 MGBB participants in the overall cohort and by race reported in the MGBB. ^c^(*n*_cases_^high-risk PRS^/*n*_controls_^high-risk PRS^, *n*_cases_^without high-risk PRS^/*n*_controls_^without high-risk PRS^). NaN, not a number.

### Prospective PRS assay

#### Sensitivity and specificity of array and imputation

The replication results above supported the development of a genotype array-based clinical assay for PRS and secondary findings from the American College of Medical Genetics and Genomics v2.0 list (ACMG SF v2.0)^24^. To determine the performance of the arrays used in the prospective assay and of the imputation pipeline, we used three reference Genome In A Bottle (GIAB) samples (NA12878, NA24385 and NA24631, Supplementary Table 5)^25^. Sensitivity and positive predictive value (PPV) for single-nucleotide variants (SNV) were > 99.7% on average, with lower performance for indels (sensitivity, 96.3%; PPV, 97.8%). Of note, although sensitivity in the ACMG SF v2.0 regions was high (96.2%), PPV was low (63.6%) due to these regions having an excess of poorly performing rare variants^26,27^.

As expected, sensitivity and PPV decreased for imputed data, especially for indels (SNV sensitivity, 98.0%; SNV PPV, 97.5%; indel sensitivity, 92.8%; indel PPV, 90.7%) (Supplementary Table 5). NA12878 was not evaluated for imputation accuracy because it is present in the imputation reference dataset from the 1000 Genomes Project and has artificially high imputation accuracy. To further evaluate imputation accuracy, we compared genome sequencing data to array data for 22 diverse samples. Analytical performance was lower in this dataset than in the GIAB high-confidence data (~3% reduction in performance for sensitivity and PPV, Supplementary Table 6).

#### Performance of prospective PRS assay

**Extended Data Fig. 8:**
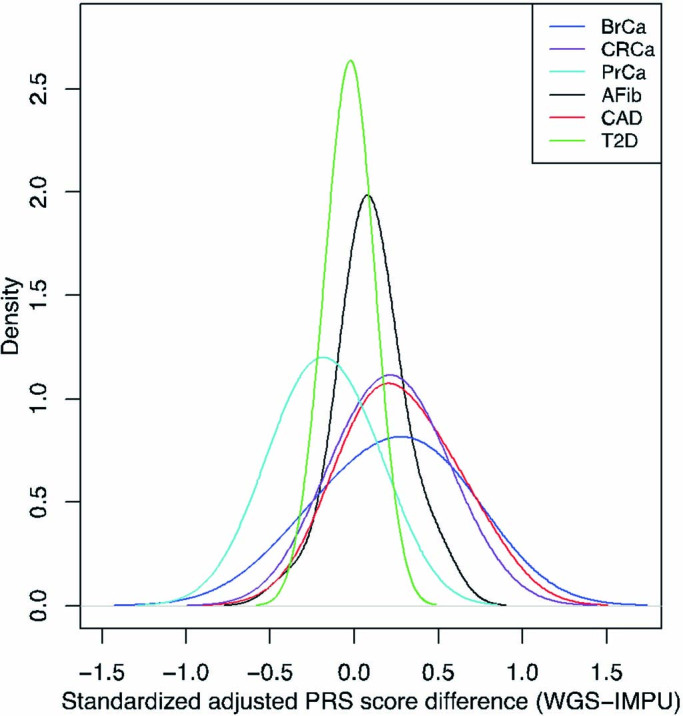
Difference in standardized, adjusted PRS between WGS and imputed genotyping arrays for 22 individual samples. The PRS_std-adj_ of 22 samples obtained from WGS and from imputed genotyping arrays are subtracted, and the distribution of the difference of the scores is plotted for each disease. Abbreviations: AFib, atrial fibrillation; BrCa, breast cancer; CAD, coronary artery disease; CRCa, colorectal cancer; IMPU, imputed genotype data; MGBB, Mass General Brigham Biobank; PrCa, prostate cancer; PRS, polygenic risk score; T2D, type 2 diabetes; WGS, whole genome sequencing.

For the GIAB samples, PRS_std-adj_ was robust across different array versions and consistent with results from whole genome sequence (WGS) data; all three GIAB samples were below the high-risk threshold (OR > 2) for all diseases in all methods (Supplementary Table 7). In evaluating the 22 samples with WGS and prospective array data, PRS_std-adj_ scores were similarly concordant, particularly for AFib, CAD and T2D (Extended Data Fig. 8). Additionally, 108/110 high-risk status classifications were concordant in this dataset (98.2% agreement; Matthews correlation coefficient, 0.84; *P* < 0.001), with the two discordant values (one in CAD and one in CRCa) being very close to the high-risk threshold (Supplementary Table 8). Finally, we compared nine individuals with high-risk PRS for 10 diseases identified in the MGBB genotyping data to their PRS risk status using the prospective assay (one individual at high risk for AFib, one individual at high risk for BrCa, three individuals at high risk for CAD, three individuals at high risk for CRCa, one individual at high risk for PrCa and one individual at high risk for T2D). All PRS categories were consistent across the two different arrays used for MGBB genotyping and for the clinical assay (Supplementary Table 9).

#### Clinical PRS report

We then developed a PRS laboratory report consistent in format and content with other clinical genetic test reports (Supplementary File 1)^28–30^. That is, it includes a description of the test performed and a prominently displayed summary of important findings and their interpretations. Subsequent sections of the report give more detail about the results, including, for each disease, general population prevalence and a brief summary of the GWAS from which the PRS was derived. Sections on methodology and literature references are at the end of the report. The report also reflects several choices made during its development. A graphic highlights in red the disease(s) for which the patient has increased polygenic disease risk, as defined by a PRS corresponding to a published OR > 2 for disease, mirroring both a common threshold from Mendelian genetics^31^ and the effect sizes for disease risk factors already considered in current clinical care^32–36^. Any PRS not categorized as high risk is described as conferring average risk. Monogenic disease variants and PRS results are reported separately, without comment on any possible interaction between a monogenic result and a relevant PRS (for example, an average-risk BrCa PRS and a pathogenic variant in *BRCA1* associated with hereditary breast and ovarian cancer). Illustrating the boundary where the role of the clinical laboratory ends (phase 2 of Fig. 1) and the role of the treating physician begins (phase 3), the laboratory report does not include information about absolute disease risk or the role of other, non-genetic factors in disease risk, and it is not directive in its recommendations for clinical management of high-risk results.

#### Clinical processes and supportive materials

In recognition that physicians and patients require additional guidance in contextualizing high-risk PRS results, the GenoVA Study has developed processes and materials to support the clinical use of PRS. A genetic counselor contacts each patient with a high-risk PRS result or monogenic disease variant to discuss the result’s health significance and offer guidance for a conversation to have with their physician. All patients and their primary care physicians receive a copy of the laboratory report, and each patient with at least one high-risk PRS result is additionally given patient-oriented educational materials about the relevant disease(s) (Supplementary File 2). The patient’s primary care physician also receives a copy of physician-oriented educational materials to support their decision-making about PRS (Supplementary File 3). Given the current state of the evidence, the physician materials note that professional guidelines do not recommend specific changes to general screening or prevention recommendations based on PRS results, but these materials are updated over the course of the study as evidence accrues to support distinct recommendations.

#### Results from the first 227 prospective samples

As of 21 October 2021, 227 GenoVA trial participants have been assayed using the prospective PRS pipeline from two primary sample types (130 blood, 97 saliva). Of these, 108 participants (48%) self-report as white race and non-Hispanic/Latinx ethnicity, and 78 (34%) identify as women. In this preliminary sample of trial enrollees, the proportions of participants whose PRS are above the study threshold for high risk are consistent with those observed in the MGBB, ranging from 5.7% for CRCa to 15.3% for PrCa (Table 2). Two actionable ACMG SF v2.0 variants have been identified and confirmed in the first 227 enrollees (*BRCA1*:NM_007294 c.2748delT (p.Asn916LysfsX84), likely pathogenic; *BRCA2*:NM_000059 c.3545_3546delTT (p.Phe1182X), pathogenic). The reporting of these results to trial participants and their physicians is underway. The study will determine whether PRS implementation affects clinical management and enables the detection of undiagnosed prevalent cases and incident cases during the observation period.

**Table 2:** Summary of PRS results from the first six batches of clinical samples in the GenoVA Study

	BrCa	CRCa	PrCa	AFib	CAD	T2D
Total analyzed, *n*	77	227	150^a^	227	227	227
Average risk, *n* (%)	67 (87.0)	214 (94.3)	127 (84.7)	203 (89.4)	211 (92.9)	210 (92.5)
High risk, *n* (%)	10 (13.0)	13 (5.7)	23 (15.3)	24 (10.6)	16 (7.1)	17 (7.5)

Results from the first 227 GenoVA participants. High-risk PRS, defined here as PRS_std-adj_ associated with OR > 2 for disease in the original publication. All other results are considered as average risk.

^a^One participant with male sex identifies as female.

## Discussion

Bridging two significant gaps between PRS development and clinical implementation, we developed a clinical genotyping array-based assay for six PRS and a process to report the results to patients and primary care physicians. The PRS were robust across multiple genotyping arrays and imputation pipelines. The distributions of unadjusted PRS varied by reported race in a large biobank, impeding clinical validation, but adjustment for population structure enabled the replication of published PRS–disease associations. These results supported the development of a population structure-adjusted pipeline for PRS calculation and reporting for individual patients, now implemented in a clinical trial of PRS testing along with patient and physician educational materials and genetic counseling support.

The development and implementation of our PRS assay and report illustrate key choices that laboratories must make in what we term phase 2 of the PRS implementation pathway. First, for each target disease, we had to choose the specific PRS to implement among multiple publicly available options (that is, PRS developed and validated by others in phase 1)^21,22^. Considerations include the performance of the PRS in both the published discovery and replication cohorts in addition to the population that the laboratory is interested in targeting. Guidelines are emerging on what defines high-quality PRS reporting^37^, and this improved transparency should help laboratories to select appropriate PRS from the many available. Second, we chose to use a genotype array-based approach instead of genome sequencing. Like genotyping, low-coverage genome sequencing technology is also relatively low cost^38^. We chose the Illumina GDA because its widespread use in the All of Us Research Program^39^, eMERGE Consortium^15^ and other projects optimizes the likelihood that it will be a well-supported genotyping platform for future improvements, and enhances the generalizability of our methods to other institutions looking to implement clinical PRS testing. Third, although published methods can adjust for population structure in large cohorts of people^40,41^, these methods are not immediately applicable for correcting a PRS for a prospectively genotyped individual patient, whose sample is at best part of a small clinically analyzed batch with insufficient data for robust population structure adjustment. Correction thus requires additional decisions about how to adjust for population structure and which reference to use. We chose to impute data against 1000 Genomes Project phase 3 data and to project each new individual patient sample onto the principal components from the MGBB. Other laboratories may choose to impute against the larger TOPMed (Trans-Omics for Precision Medicine) population^42^, although issues of genome build discrepancy and regulatory prohibition against sending patient data to external research servers are limitations. Fourth, once a platform is selected, a clinical laboratory must determine the benchmarks that define an analytically valid PRS assay. We chose to verify the PRS performance in our laboratory to determine the appropriate parameters for our assay; calculate the analytical performance of the genotyping array and imputation pipeline using both well-characterized reference samples and individual level genome data; and calculate the robustness and performance of the PRS using genome data and multiple array platforms from both reference and individual samples. This multi-step approach helped ensure the accuracy of the data going into the PRS as well as the final performance of the PRS itself.

We also made numerous choices in how to report PRS results and interpretations to patients and physicians. We decided to report a dichotomous PRS interpretation (that is, high risk versus average risk) instead of a continuous result (for example, percentile rank, relative risk or absolute risk). We have previously described the trade-offs of these approaches, including the need for actionability thresholds; transparency about the limitations of PRS, particularly in underrepresented populations; and the absence of validated predictions models that incorporate both PRS and other clinical risk factors^43^. For the GenoVA Study we favored a dichotomous result to indicate a possible clinical action threshold to the treating physician. We chose OR > 2 to define high polygenic risk, consistent with effect sizes of traditional risk factors considered for the target diseases^32–36^. Another laboratory may use the methods we describe to produce measures of continuous risk or of categorical risk at different thresholds thought to be clinically meaningful, which will probably vary among the diseases for which they choose to implement PRS. Estimating absolute disease risk (for example, with the BOADICEA model for breast cancer^44^ or the Pooled Cohort Equations for atherosclerotic cardiovascular disease^45^) may be considered the gold standard for risk stratification, but validated absolute risk models are not available for most diseases and require patient information (for example, mammographic breast density and blood pressure) that is often unavailable to the interpreting laboratory. Drawing on other examples from primary care, we chose not to include directive clinical recommendations on the PRS laboratory report itself, instead assigning such activities to phase 3 of the PRS implementation pathway, supported by informational materials and genetic counseling support. We note that, for example, although a laboratory reports the results of a patient’s low-density lipoprotein cholesterol and reference range for the assay, it is the treating physician who contextualizes that result with the patient’s other characteristics to decide whether to offer cholesterol-lowering therapy.

The question of how to support physician management of PRS results without under- or overselling the potential benefits of PRS is controversial, given the lack of prospective data showing that the clinical use of PRS improves patient outcomes. In this early era of PRS implementation, the most prudent course of action is probably to develop educational and consultant resources, such as those used in the GenoVA Study, to present transparently the evidence for and limitations of PRS interpretations without being overly prescriptive in their recommendations. Given the participant age range and choice of diseases in the GenoVA Study, we anticipate that most physician actions will fall within already clinically acceptable practices (for example, more frequent hemoglobin A1c screening for T2D or favoring colonoscopy screening over fecal immunochemical testing for CRCa screening). Stronger evidence of benefit will be needed to justify actions that deviate more significantly from accepted practice, such as screening starting at much younger ages or requiring more invasive or expensive procedures. As they do in all areas of medicine, physicians will need to use available evidence and clinical judgment to make the best decisions with their patients. The GenoVA Study is collecting data on what physicians do with PRS results and their preferences for how they can be supported in this decision-making.

Although other laboratories are developing PRS assays in both clinical and research settings and have reported the aggregate performance of these PRS in a population, including biobanks or customers of direct-to-consumer companies^15,38,46–48^, none has described the development and validation of a clinical, population structure-adjusted assay for prospectively tested individuals. Although the eMERGE consortium and other studies are actively developing trans-ancestry PRS for a number of common diseases^15,49^, we report, here, a single clinical assay for population structure-adjusted PRS for multiple diseases. And while other laboratories may make different decisions about the number of disease PRS they choose to implement, whether and how to compare the performance of multiple available PRS for each disease, and the format of the clinical PRS report, our work provides a framework for how a laboratory can clinically validate and implement a prospective PRS suitable for an individual patient.

Much has been written about the reduced validity of most PRS in populations of non-European ancestry, due to their use of non-causal loci and effect sizes from GWAS in predominantly European discovery cohorts^19,20,50,51^. As we await larger datasets from more diverse populations and the methodological advances that will improve the performance of trans-ancestry PRS^10,15,49^, a clinical laboratory looking to develop a PRS assay for a given disease has the following options: (1) postpone implementation, as done by some commercial laboratories;^52,53^ (2) implement separate ancestry-specific published PRS only in those ancestral groups from which they were derived and validated; or (3) implement a single PRS that aims for applicability across ancestry groups and report transparently any applicable limitations in the underlying evidence and its interpretation for specific individuals or ancestral groups. Because the second option requires the assignment of an individual patient to a specific ancestry group, either before or during PRS analysis, and, problematically, risks the inequitable provision of PRS to some populations but not to others, we chose the third option for the GenoVA Study and implemented a single method of adjustment for population structure. After doing so, we observed that the chosen PRS threshold corresponding to OR > 2 generally identified subjects at higher risk of disease across reported race in the MGBB replication cohort. The magnitude and precision of this effect did vary by reported race, probably due to two factors: small numbers of MGBB cases for certain diseases in certain racial groups; and real differences in the ability of these PRS to correlate with disease risk in non-European ancestry groups, as has been observed even in well-developed trans-ancestry PRS^10,54^. Methodological advances that leverage local ancestry or GWAS summary statistics from multiple diverse populations will improve the performance of PRS across ancestry groups^55,56^. In the meantime, we have developed a clinically validated PRS assay, the application of which in diverse ancestry groups is defensible but the results of which, nonetheless, have limitations. These limitations are clearly presented in a clinical laboratory report (phase 2), which can then be contextualized by the physician for each individual (phase 3). Applying population-level data to individual patient care represents both the science and art of medical practice, particularly when the individual patient is not well represented in the available data^57,58^.

In conclusion, data from increasingly larger and more diverse populations, coupled with computational advances, are propelling PRS into consideration for clinical implementation. We have shown that laboratory assay development and PRS reporting to patients and physicians are feasible (but non-trivial) next phases in PRS implementation. As the performance of PRS continues to improve, particularly for individuals of underrepresented ancestry groups, the implementation processes we describe can serve as generalizable models for laboratories and health systems looking to realize the potential of PRS for improved patient health.

## Methods

### Selection of PRS for implementation

We identified large GWAS for the six target diseases for which the summary statistics (base files with alleles and weights) were freely available from the Polygenic Score (PGS) Catalog^21^ (AFib, CAD, T2D, BrCa) or the Cancer PRSWeb (CRCa, PrCa)^22^ as of 26 December 2019. For the three cardiometabolic diseases (AFib, CAD and T2D) we chose the PRS derived from the UK Biobank in Khera et al. 2018 (ref. ^7^): for AFib, the PGS Catalog Publication (PGP) ID is PGP000006 and the PGS ID is PGS000016; for CAD, the PGP ID is PGP000006 and the PGS ID is PGS000013; and for T2D the PGP ID is PGP000006 and the PGS ID is PGS000014. For the three cancers we chose PRS derived from the largest published GWAS at the time: for BrCa we used Michailidou et al. 2017 and Mavaddat et al. 2019 (PGS ID = PGS000007, PGP ID = PGP000002) (ref. ^59,60^); for CRCa we used Huyghe et al. 2019 (PRSWEB_PHECODE153_CRC-Huyghe_PT_MGI_20191112, PRS tuning parameter: 3.98107170553497e-07) (ref.^23^); and for PrCa we used Schumacher et al. 2018 (PRSWEB_PHECODE185_Pca- PRACTICAL_LASSOSUM_MGI_20191112, PRS tuning parameter: s0.5_Lambda0.00695192796177561) (ref. ^61^).

### Replication of published PRS

#### Population and sample

Given that the GenoVA Study is enrolling participants from eastern Massachusetts, USA, we used data from the Mass General Brigham (formerly Partners Healthcare) Biobank (MGBB)^62^ to evaluate the performance of the selected PRS in a similar population and workflow for our study and assay. MGBB participants were not included in the published derivation and validation studies for the PRS used. In brief, MGBB was launched in 2010 with the initial goal of collecting DNA, plasma, and serum samples from 75,000 patients from Brigham and Women’s Hospital, Massachusetts General Hospital, and other MGBB-affiliated healthcare facilities, and obtaining patient consent for the linkage between biospecimen data, medical record data and survey data. We use the terms ‘race’ and ‘ethnicity’ to refer to social constructs often used in healthcare operations and biomedical research to evaluate and address disparities between populations. Racial categories of participants in the MGBB (for example, white or Asian) are derived from electronic health record (EHR) data. For the present analysis we collapsed reported race in MGBB into four categories: Asian, Black, white, and other/unknown. Race and ethnicity of GenoVA Study participants were collected through EHR data and self-report and categorized using the five racial categories (American Indian or Alaska Native, Asian, Black or African American, Native Hawaiian or Other Pacific Islander, and white) and two ethnic categories (Hispanic/Latinx and Not Hispanic/Latinx) required by US federal data collection standards. We use the term ‘ancestry’ to describe the genetic construct describing inheritance of variants from global ancestral populations.

#### Disease phenotyping

We used validated computed phenotypes from MGBB to define case and control status for each of the six diseases (Supplementary Table 1). Validated MGB phenotypes are available for CAD (PPV = 95%), AFib (PPV = 94%), T2D (PPV = 95%), and colorectal (PPV = 100%), breast (PPV = 95%) and prostate cancer (PPV = 100%)^63–65^. For each disease, ‘caseness’ was defined as prevalent disease on 16 December 2019. For subgroup analyses, participant age was determined on 16 December 2019 or at death, if earlier. Only women and men were assigned case or control status for breast and prostate cancer, respectively.

#### Genotyping and imputation

We used genotype data from the 36,423 MGBB participants with available genotyping data as of 16 December 2019. Genotyping was performed using standard processing described previously on one of three Illumina Infinium genotyping arrays: (1) a pre-release version developed by the Multi-Ethnic Genotyping Array Consortium (Multi-Ethnic Genotyping Array (MEGA), *n* = 4,924); (2) an expanded version of this pre-commercial array (Expanded Multi-Ethnic Genotyping Array (MEGAEX), *n* = 5,345); and (3) the final commercial version (Multi-Ethnic Global (MEG), *n* = 26,157). The MEGA, MEGAEX and MEG arrays consisted of 1.39, 1.74 and 1.78 million probes, respectively^66^. For MEGA and MEGAEX data, only probes found in the commercial version of the array (MEG) were used in the present analysis. Quality control for the genotyping requires samples to have at least a 99% call rate and concordant sex between the EHR and what is computed from the array data. We used existing MGBB imputed data generated by batching sets of ~5,000 participants and imputing against the 1000 Genomes Project phase 3 data using the Michigan Imputation Server^67^ (https://imputationserver.sph.umich.edu/index.html#!), with ShapeIT (v2.r790) (ref. ^68^) used for phasing and Minimac3 used for imputation with default settings. Sets of imputed variants were compared with the base files for each PRS to ensure sufficient representation of probes (Supplementary Table 2) (ref. ^67^).

#### Calculation of PRS and adjustment for population structure

Unadjusted raw PRS (PRS_raw_) for each disease were calculated using PLINK (v.2.0a) by taking the product of the count of risk alleles and the risk allele weight at each locus in the PRS and then summing across available risk loci. The loci included in each PRS, the risk alleles and the corresponding weights were downloaded from the PGS Catalog or Cancer PRSWeb. A population structure-adjusted PRS was calculated for each disease, using a previously described approach^40^ implementing principal components analysis to compute adjusted residualized PRS for each disease. Principal components were calculated using all genotyped MGBB participants and a set of 16,385 of 16,443 previously reported ancestry-informative SNPs^69^. For each disease we then fit a linear model for PRS_raw_ as a function of the first four principal components in controls for that disease (PRS_raw_ ~ PC1 + PC2 + PC3 + PC4) in R (v.4.0.3). We then applied this model to calculate a predicted PRS (PRS_pred_) for each disease in all cases and controls. Residualized, population structure-adjusted PRS (PRS_adj_) were then computed for each individual for each disease as the difference between the raw and the predicted PRS (PRS_raw_ − PRS_pred_). For PRS_raw_, values were standardized (PRS_std-raw_) using the mean and standard deviation in the MGBB of the PRS_raw_ values (Supplementary Table 3). Similarly, PRS_std-adj_ was computed using the mean and standard deviation in the MGBB of the PRS_adj_ values (Supplementary Table 3). The distributions of PRS_std-raw_ and PRS_std-adj_ by genotype array, sex, age deciles and reported race were compared among all subjects using the density function in R (v.4.0.3).

#### PRS–disease association

The association of PRS_std-adj_ with the odds of disease was replicated in MGBB participants using the six disease phenotypes described above. For each PRS and disease, odds of disease (*n*_cases_/n_controls_) were calculated for each of 50 PRS quantiles. For race-stratified analyses, PRS deciles were used if too few cases were available for analysis across 50 quantiles. To visualize the PRS–disease associations, we plotted the log(odds) of disease against the mean PRS_std-adj_ in each quantile. Correlation was measured with Pearson correlation coefficients using RStudio (v.1.1.383) with R (v.4.0.3).

#### PRS threshold for high risk

We set a predicted polygenic OR > 2 to identify individuals at high polygenic risk for each disease, mirroring both a common threshold from Mendelian genetics^31^ and the effect sizes for disease risk factors already considered in current clinical care^32–36^. To operationalize this OR > 2 threshold, we compared standardized PRS *Z* scores for each individual to a disease-specific cut off 𝛕, based on previously published estimates of the change in odds of disease per standard deviation change in the PRS (Supplementary Table 3). Specifically, 𝛕 = ln(2)/ln(OR_s.d._), where 2 is the target OR threshold defining high risk and OR_s.d._ is the estimated multiplicative change in odds per standard deviation change in the PRS. Assuming that the published OR_s.d._ accurately captures the relationship between PRS and disease, the odds of disease for individuals with standardized PRS *Z* score = 𝛕 are twofold that of individuals with a median PRS *Z* score. These standardized PRS thresholds were used to assign individual patients to risk categories as described below (PRS calculation for clinical assay for individual samples).

### Clinical PRS assay for individual samples

Based on the results of the above methods, we developed and validated a genotype array-based clinical assay for PRS, in addition to secondary findings from the ACMG v2.0 list (ACMG SF v2.0, Fig. 1)^24^. We include additional variants identified by the ACMG or other organizations as important secondary findings as updated recommendations accrue^70^.

#### Validation samples

Replicates of each of three reference samples from GIAB^25^ maintained by the National Institute of Standards and Technology were included in the validation assay: NA12878 × 9, NA24631 × 6 and NA24385 × 6. Analytical performance (sensitivity and PPV for presence or absence of variant sites) was determined in the benchmarking regions (v3.3.2). In addition, we included 22 samples with polymerase chain reaction-free genome sequencing data (described below) and 9 samples with high-risk PRS for one of the six diseases as determined by the MGBB data, including one individual with high-risk PRS for two diseases. To test the sensitivity of the secondary finding analysis, we genotyped 20 samples with previously identified pathogenic or likely pathogenic variants in the ACMG SF v2.0 list.

#### Genotyping and imputation

Validation samples were genotyped according to manufacturer-standard workflows on either a pre-commercial release of the Illumina Global Diversity Array (GDA-PC) or the final commercial release of the Global Diversity Array (GDA). The Illumina-specific files containing called genotypes in AA/AB/BB format (GTC files) generated by genotype array were converted to variant call format (VCF) using a modified version of the gtc2vcf script from Illumina. All samples required an overall call rate of greater than 98.5%. Imputation was performed using updated software, with EAGLE v2.4.1 (ref. ^71^) for phasing and Minimac4 (ref. ^67^) for imputation using the 1000 Genomes Project phase 3 dataset. Importantly, monomorphic sites were not removed during the imputation process due to the small batch sizes used in the prospective assay.

#### PRS calculation for clinical assay for individual samples

PRS_raw_ was calculated for each sample as described above. To determine PRS_adj_, unadjusted PRS (PRS_raw_) were first calculated for each individual sample as described for the overall MGBB cohort. For each individual, the eigenvariable, eigenvalue and frequency output from the MGBB principal components analysis were used to project each new individual sample onto the MGBB principal components, using the following command in PLINK v.2.0a:^72^


plink2 —pfile individual_data —read-freq ref_pcs.acount —score ref_pcs.eigenvec.allele 2 5 header-read no-mean-imputation variance-standardize —score-col-nums 6-15 —out new_projection


The resulting projected principal components were then scaled to match the MGBB principal components by taking the square root of the eigenvalue and then multiplying by 2. The scaled principal components (PCs) were fitted into the linear model for each disease developed in the MGBB data to obtain PRS_pred_:

BrCa: PRSpred = 17.609341*PC1 − 4.146935*PC2 + 5.335144*PC3 + 3.833931*PC4 − 0.421679

CRCa: PRSpred = −13.659121*PC1 + 6.411109*PC2 − 2.483703*PC3 − 6.869127*PC4 + 6.131384

PrCa: PRSpred = 23.441147*PC1 + 13.724771*PC2 − 9.528270*PC3 + 4.118756*PC4 + 11.506243

AFib: PRSpred = 9.6269881*PC1 − 3.2878238*PC2 − 6.6519006*PC3 − 3.0149108*PC4 + 32.4067610

CAD: PRSpred = −6.1974327*PC1 − 3.6757094*PC2 − 1.3488677*PC3 − 1.3490566*PC4 + 18.0582457

T2D: PRSpred = 26.4700782*PC1 − 7.4283370*PC2 + 9.3782116*PC3 + 1.6994457*PC4 + 55.6998719

PRS_adj_ was then calculated as the difference between PRS_raw_ and PRS_pred_. Standardized, adjusted PRS values (PRS_std-adj_) were calculated using the mean and standard deviation of PRS_adj_ in MGBB and compared against the PRS threshold corresponding to OR > 2 as determined from the original publications (Supplementary Table 3). Any PRS_std-adj_ result above the PRS threshold corresponding to OR > 2 was categorized as high polygenic risk.

#### Genome sequencing

We selected 22 diverse samples that had previously undergone clinical whole genome sequencing to determine the robustness of PRS across different platforms. Genome sequencing was performed at the Clinical Research Sequencing Platform of the Broad Institute using polymerase chain reaction-free library construction and sequencing on an Illumina NovaSeq with two 150 bp paired-end reads with ≥95% of bases covered at ≥20-fold. Reads were aligned to GRCh37 using the Burrows–Wheeler Aligner (BWA v.0.7.15)^73^ and variant calls were made using HaplotypeCaller from the Genomic Analysis Tool Kit (GATK v.4.0.3.0)^74,75^. PRS_raw_, PRS_std-raw_, PRS_adj_ and PRS_std-adj_ were calculated as above for the other prospective samples. As stated above, these 22 samples were also analyzed on the GDA-PC array to compare PRS between genome sequencing and array. The difference between the sequence-based and array-based PRS were visualized, and dichotomous risk classifications were formally compared using the Matthews correlation coefficient^76^.

#### Identification of actionable variants associated with monogenic disease

Variants from the original genotyping VCF were annotated and filtered to the 59 genes suggested for screening of secondary findings as recommended by the ACMG (ACMG SF v2.0)^24^ to find: (1) variants previously identified as disease causing by the MGB Laboratory for Molecular Medicine; (2) variants classified as pathogenic or likely pathogenic in ClinVar with a minor allele frequency (MAF) < 0.1%; (3) variants classified as a disease-causing mutation in the Human Gene Mutation Database with a MAF < 0.03%; and (4) loss-of-function variants (nonsense, frameshift, canonical splice-site, and initiating methionine variants) with a MAF < 0.1% in genes in which that is a disease mechanism. Clinical variant classification was carried out in accordance with the criteria set by the guidelines by the ACMG and the Association of Molecular Pathology^77^, with disease-specific modifications as recommended by the Clinical Genome Resource Expert Panels^78^.

### Prospectively enrolled trial participants

The assay described above is now in use in the ongoing GenoVA Study randomized trial of clinical PRS (ClinicalTrials.gov identifier: NCT04331535), in which eligible participants are patients of the VA Boston Healthcare System, aged 50–70 years, without known diagnoses of the six target diseases. Enrollees provide a clinical blood or saliva sample for analysis at the Laboratory for Molecular Medicine.

### Ethics declaration

Analyses of the genomic and MGBB samples and data have been reviewed and approved by the Mass General Brigham institutional review board (2019P001933). Analyses for the prospective pipeline, including the use of prior clinical samples, were conducted under the Mass General Brigham institutional review board (2004P001056); all individuals with clinical testing, including those with genome sequencing data, gave consent for clinical testing, and all individual data were de-identified. The GenoVA Study is approved by the VA Boston Healthcare System (no. 3241) and Harvard Medical School institutional review board (IRB19-0594), and all enrollees provided written informed consent.

### Reporting Summary

Further information on research design is available in the Nature Research Reporting Summary linked to this article.

## Online content

Any methods, additional references, Nature Research reporting summaries, source data, extended data, supplementary information, acknowledgements, peer review information; details of author contributions and competing interests; and statements of data and code availability are available at 10.1038/s41591-022-01767-6.

## Supplementary information

Supplementary Information Supplementary Tables 1–9 and Files 1–3.

Reporting Summary

## Data Availability

The majority of the MGBB genotyped samples are deposited in dbGAP as part of the eMERGE consortium, phase 3 (https://www.ncbi.nlm.nih.gov/projects/gap/cgi-bin/study.cgi?study_id=phs001584.v2.p2). Additional MGBB data were accessed under institutional review board protocol for this current study and are not publicly available due to restrictions on the data. Data from the GenoVA Study trial will be made publicly available after study completion. The 1000 Genomes Project phase 3 dataset used in this study was v5a and was downloaded from ftp://ftp.1000genomes.ebi.ac.uk/vol1/ftp/release/20130502/.

## Extended data

are available for this paper at 10.1038/s41591-022-01767-6.

## Code availability

The code used to adjust the PRS for population structure is available for download here: https://github.com/MGB-Personalized-Medicine/PRS-adjustment.
