# Supplementary material for: Development of a clinical polygenic risk score assay and reporting workflow
Source: Nat Med. Author manuscript; Available in PMC 2022 May 20. (PMC9117136; doi:10.1038/s41591-022-01767-6)
Supplement: Supplementary Information [file NIHMS1801218-supplement-Supplementary_Information.pdf]

nature portfolio

https://doi.org/10.1038/s41591-022-01767-6

Supplementary information

Development of a clinical polygenic risk score assay and reporting workflow

In the format provided by the
authors and unedited

SUPPLEMENTARY INFORMATION

Supplemental Table 1. Disease phenotypes and counts in MGB Biobank.

Supplemental Table 2: Counts of constituent SNPs from PRS base files and from 4 genotype arrays after imputation for 6 diseases.

Supplemental Table 3. PRS thresholds corresponding to published OR>2.

Supplemental Table 4. Prevalence and disease associations of high-risk PRS for 6 diseases in MGB Biobank, before adjustment for population structure.

Supplemental Table 5. Analytical performance of genotyping arrays and imputed data for three Genome in a Bottle samples.

Supplemental Table 6. Analytical performance of genotyping arrays and imputed data for 22 samples with WGS.

Supplemental Table 7. Standardized, adjusted PRS scores of three Genome in a Bottle samples compared between genome sequencing and genotyping arrays.
Supplemental Table 8. Comparison of standardized, adjusted PRS between WGS and genotyping arrays for 22 samples.

Supplemental Table 9. Comparison between standardized, adjusted PRS scores from MGBB data and prospective assay for diseases in nine samples with high risk.

Supplemental File 1. Example of PRS report

Supplemental File 2. Disease-specific PRS educational materials for patients

Supplemental File 3. Disease-specific PRS educational materials for physicians

Supplemental Table 1. Disease phenotypes and counts in MGB Biobank

Disease	PPV(%)	Controls	Cases	Total
BrCa	95	17949	1770	19719
CRCa	100	36031	392	36423
PrCa	100	13783	2919	16702
AFib	94	33691	2732	36423
CAD	95	32869	3554	36423
T2D	95	33060	3363	36423

Positive predictive values (PPV) for computed disease phenotypes in MGBB, as reported previously (see main text). Also shown are the counts of controls and cases, limited to women and men for BrCa and PrCa, respectively. Abbreviations: AFib, atrial fibrillation; BrCa, breast cancer; CAD, coronary artery disease; CRCa, colorectal cancer; MGB, Mass General Brigham Biobank; PrCa, prostate cancer; T2D, type 2 diabetes.

Supplemental Table 2. Counts of constituent SNPs from PRS base files and from 4 genotype arrays after imputation

Chip	BrCa	CRCA	PrCa	AFib	CAD	T2D
Base file	3820	81	147	6730541	6630150	6917436
MEGA	3818	80	140	6730205	6630033	6917170
MEGAEX	3818	80	140	6730106	6630095	6917118
MEG	3819	80	140	6730145	6630016	6917080
GDA_early_release	3818	80	140	6730541	6630150	6917436
GDA_commercial	3818	80	140	6730541	6630150	6917436

Comparison of the PRS model for each disease and the sites available from imputation data among different arrays. Abbreviations: MEGA, Multi-Ethnic Genotyping Array; MEGAEX, Expanded Multi-Ethnic Genotyping Array; MEG, Multi-Ethnic Global array; GDA_EA, Global Diversity Array, Early Access; GDA, Global Diversity Array; BrCa, breast cancer; CRCa, colorectal cancer; PrCa, prostate cancer; AFib, atrial fibrillation; CAD, coronary artery; T2D, type 2 diabetes mellitus.

Supplemental Table 3. PRS thresholds corresponding to published OR>2

Disease	Change in OR per SD change in PRS	Z cutoff for OR>2	Source	Mean (SD) PRS_{raw} in MGBB	Mean (SD) PRS_{adj} in MGBB
BrCa	1.66	1.37	Table 2 in Mavaddat 2019	-0.3994271 (0.4717565)	0.01712425 (0.4607471)
CRCa	1.54	1.61	Supplemental Table 16 in Huyghe 2019	6.132968 (0.4337869)	0.001579539 (0.424732)
PrCa	1.86	1.12	Supplemental Table 18 in Schumacher 2018	11.53247 (0.6906457)	0.03554762 (0.6770496)
AFib	1.63	1.42	Supplemental Table 2 in Khera 2018	32.41001 (0.1241203)	0.003250882 (0.1056579)
CAD	1.72	1.28	Supplemental Table 1 in Khera 2018	18.06105 (0.09994096)	0.002804128 (0.09180318)
T2D	1.65	1.38	Supplemental Table 3 in Khera 2018	55.70348 (0.1893828)	0.003617606 (0.1123435)

Also included are the mean and standard deviation (SD) of raw (PRS_{raw}) and adjusted PRS (PRS_{adj}) as determined in MGBB and used to standardize the PRS. Abbreviations: AFib, atrial fibrillation; BrCa, breast cancer; CAD, coronary artery disease; CRCa, colorectal cancer; MGBB, Mass General Brigham Biobank; PrCa, prostate cancer; PRS, polygenic risk score; T2D, type 2 diabetes.

Supplemental Table 4. Prevalence and disease associations of high-risk PRS for 6 diseases in MGB Biobank overall and by reported race, before adjustment for population structure

Disease	High risk (%)	OR Overall	OR White	OR Black	OR Asian	OR Other/Unknown
BrCa	8.6	1.99<br>[1.72, 2.30]<br>(252/1435, 1461/16571)	2.42<br>[2.07, 2.83]<br>(223/927, 1365/13724)	1.39<br>[0.73, 2.67]<br>(13/217, 37/860)	0.84<br>[0.24, 2.90]<br>(3/61, 22/377)	2.46<br>[1.29, 4.70]<br>(13/230, 37/1610)
CRCa	5.3	2.45<br>[1.8, 3.34]<br>(47/1896, 345/34134)	2.30<br>[1.67, 3.17]<br>(44/1768, 309/28595)	4.15<br>[0.53, 32.41]<br>(1/25, 17/1764)	0<br>[0, NaN]<br>(0/13, 7/766)	5.57<br>[1.23, 25.26]<br>(2/90, 12/3009)
PrCa	13.2	2.13<br>[1.91, 2.39]<br>(486/1711, 1705/12800)	2.30<br>[2.04, 2.59]<br>(431/1322, 1581/11143)	1.71<br>[1.09, 2.69]<br>(42/206, 46/386)	0<br>[0, NaN]<br>(0/19, 8/296)	1.10<br>[0.6, 2.04]<br>(13/164, 70/975)
AFib	8.4	1.53<br>[1.36, 1.73]<br>(326/2738, 2406/30952)	2.59<br>[2.25, 2.99]<br>(251/1153, 2272/27040)	1.26<br>[0.80, 1.99]<br>(45/871, 35/856)	1.49<br>[0.60, 3.69]<br>(12/384, 8/382)	1.60<br>[0.95, 2.69]<br>(18/330, 91/2674)
CAD	9.2	1.88<br>[1.7, 2.07]<br>(532/2820, 3021/30049)	1.94<br>[1.74, 2.15]<br>(494/2387, 2689/25146)	2.47<br>[0.70, 8.69]<br>(3/14, 143/1647)	2.12<br>[1.11, 4.05]<br>(17/193, 23/553)	1.30<br>[0.78, 2.15]<br>(18/226, 166/2703)
T2D	9.6	2.53<br>[2.31, 2.78]<br>(649/2850, 2714/30209)	2.65<br>[2.11, 3.31]<br>(97/419, 2429/27771)	2.58<br>[1.6, 4.15]<br>(356/1250, 20/181)	1.01<br>[0.55, 1.87]<br>(41/533, 15/197)	1.97<br>[1.58, 2.45]<br>(155/648, 250/2060)

High-risk PRS, defined here as an unadjusted PRS (PRS_{raw}) associated with OR>2 for disease in the original publication. OR shown are the observed OR [95% CI] among up to 36,423 MGBB participants in the overall cohort and by reported race. Data below each OR take the format (n cases with high-risk PRS / n controls with high-risk PRS, n cases without high-risk PRS / n controls without high-risk PRS). Second column shows the proportion of MGBB participants exceeding the literature-derived OR>2 threshold for each disease. Abbreviations: AFib, atrial fibrillation; BrCa, breast cancer; CAD, coronary artery disease; CRCa, colorectal cancer; MGBB, Mass General Brigham Biobank; NaN, not a number; OR, odds ratio; PrCa, prostate cancer; PRS, polygenic risk score; T2D, type 2 diabetes.

Supplemental Table 5. Analytical performance of genotyping arrays and imputed data for three Genome in a Bottle samples

Sample	ROI	RAW				IMPUTED			
		SNV		INDEL		SNV		INDEL	
		Sens(%)	PPV(%)	Sens(%)	PPV(%)	Sens(%)	PPV(%)	Sens(%)	PPV(%)
NA12878	NIST_HC	99.42	99.39	99.58	94.54	98.30	98.71	91.35	93.04
	ACMG59	95.83	63.45	#	#	N/A	N/A	N/A	N/A
NA24385	NIST_HC	99.45	99.42	98.21	97.40	96.50	97.25	89.75	92.28
	ACMG59	96.84	63.01	#	#	N/A	N/A	N/A	N/A
NA24631	NIST_HC	99.25	99.27	98.06	93.79	96.36	97.21	89.52	92.14
	ACMG59	95.83	51.98	#	#	N/A	N/A	N/A	N/A

The sensitivity and positive predictive value (PPV) of three Genome in a Bottle (GIAB) Samples for both genotyping data and variants imputed from the genotyping array using 1000 Genomes Project phase 3 data. Metrics are shown for single nucleotide variants (SNVs) and insertion/deletion events (indels) separately. Values are calculated within two regions of interests (ROI): 1) NIST: high confidence regions as determined by the GIAB consortium, and 2) ACMG SF v2.0: the NIST region subsetted to the 59 genes screened for secondary findings. Secondary findings were analyzed only in genotyping data, hence not applicable (N/A) for imputed data. Abbreviations: ACMG, American College of Medical Genetics and Genomics; NIST, National Institute of Standards and Technology.

Supplemental Table 6. Analytical performance of genotyping arrays and imputed data for 22 samples with WGS

	RAW				IMPUTED			
	SNV		INDEL		SNV		INDEL	
	Sens(%)	PPV(%)	Sens(%)	PPV(%)	Sens(%)	PPV(%)	Sens(%)	PPV(%)
Mean	99.28	98.60	96.99	92.50	95.87	93.06	88.59	87.42
SD	0.15	0.47	1.58	3.02	1.11	2.98	1.55	3.65

The mean and standard deviation (SD) for sensitivity (Sens) and positive predictive value (PPV) of 22 samples for both genotyping data and variants imputed from the genotyping array using 1000 Genomes Project phase 3 data. Truth defined as variant calls from whole genome sequencing (WGS). Metrics are shown for single nucleotide variants (SNVs) and insertion/deletion events (indels) separately.

Supplemental Table 7. Standardized, adjusted PRS scores of three Genome in a Bottle samples compared between genome sequencing and genotyping arrays

Sample name	Sample number	Method	BrCa	CRCa	PrCa	AFib	CAD	T2D
NA12878	6	WGS	0.0046	0.1936	N/A	-1.5652	-0.7087	-0.1282
	2	MEG	-0.2425	0.0527	N/A	-1.5501	-0.8127	-0.0756
	9	GDA_EA	-0.3183	0.0567	N/A	-1.5634	-0.5685	-0.0002
	2	GDA	-0.4407	0.0529	N/A	-1.5738	-0.7613	0.0339
NA24631	3	WGS	N/A	-0.0335	0.5378	-0.1916	1.2640	-1.4254
	2	MEG	N/A	0.0066	0.7879	-0.5102	1.2694	-1.3991
	6	GDA_EA	N/A	0.0499	0.5433	-0.5026	0.6418	-0.4139
	1	GDA	N/A	0.0503	0.5451	-1.4717	1.2727	-1.2834
NA24385	3	WGS	N/A	1.1379	-0.0092	-1.3015	0.2716	0.4582
	2	MEG	N/A	1.1379	-0.2275	-1.1228	0.2453	0.6089
	6	GDA_EA	N/A	0.9535	-0.2027	-1.5265	-0.0329	0.6133
	1	GDA	N/A	1.0942	0.0839	-1.4717	0.2333	0.7062

Average standardized, adjusted PRS ($PRS_{std\text{-}adj}$) for 3 Genome in a Bottle (GIAB) samples calculated from whole genome sequencing (WGS) and imputed data from 3 different genotyping arrays. Abbreviations: MEG, Multi-Ethnic Global array; GDA_EA, Global Diversity Array - Early Access; GDA, Global Diversity Array; BrCa, breast cancer; CRCa, colorectal cancer; PrCa, prostate cancer; AFib, atrial fibrillation; CAD, coronary artery; T2D, type 2 diabetes mellitus.

Supplemental Table 8. Comparison of standardized, adjusted PRS between WGS and genotyping arrays for 22 samples

		BrCa		CRCa		PrCa		AF		CAD		T2D	
Sample	Race	WGS	IMPU	WGS	IMPU	WGS	IMPU	WGS	IMPU	WGS	IMPU	WGS	IMPU
1	U	-1.8867	-1.9602	0.5725	0.0541	NA	NA	-0.4783	-0.7542	-0.4876	-0.7174	-0.6895	-0.5798
2	U	-0.7455	-0.8262	-0.7147	-0.9928	NA	NA	0.1293	0.0401	2.0022	1.4268	-0.8434	-0.7031
3	W	-0.5974	-0.2353	-0.5253	-0.8340	NA	NA	0.8934	0.8328	0.4514	0.1264	-0.7919	-0.5170
4	W	1.2894	0.6875	-0.2244	-0.2234	NA	NA	-1.1754	-1.2732	-0.0191	-0.3584	1.2915	1.2000
5	U	NA	NA	-0.2838	-0.4996	-1.7625	-1.5304	-0.5714	-1.0795	0.5459	0.4484	-0.0951	0.1091
6	W	NA	NA	-1.5450	-1.9562	-0.9071	-0.5058	1.9406	1.7445	1.1350	0.4059	0.7059	0.7560
7	W	NA	NA	1.2426	1.4942	-1.0596	-1.1552	1.1059	0.8242	-1.8147	-1.8903	-2.1168	-2.1413
8	W	-0.0807	0.0807	0.4206	0.5619	NA	NA	2.5232	2.3368	-0.3928	-0.5078	-1.1037	-0.9188
9	B	NA	NA	0.9960	0.7864	-1.0988	-1.1681	-0.0085	-0.1192	-0.6908	-0.8890	-0.2007	-0.2609
10	W	NA	NA	0.9896	0.8868	-0.4751	-0.5790	0.0136	-0.0659	-0.8545	-1.3623	-0.3380	-0.5170
11	B	2.0035	1.6508	1.7024	1.4845	NA	NA	1.1716	1.1790	-1.7253	-1.8242	0.5494	0.5569
12	W	NA	NA	0.9835	0.5430	-1.4750	-1.3309	-0.2462	-0.2993	1.0579	0.6188	-0.4822	-0.4927
13	B	0.8077	0.4828	0.5556	0.3867	NA	NA	0.4374	0.8153	1.1033	0.5647	-0.1753	-0.2103
14	B	-1.3930	-1.6669	0.9154	0.8493	NA	NA	-0.8602	-0.7490	-0.3770	-1.0892	-0.4187	-0.3697
15	B	NA	NA	0.5894	0.2448	-0.3288	-0.0110	0.3465	0.2209	-1.1982	-1.2453	1.3053	1.3296
16	B	NA	NA	0.6200	0.6782	-1.3133	-1.0626	0.5053	0.4669	-1.9614	-1.8994	1.0686	1.1810
17	B	-1.9561	-1.8142	0.3151	-0.3847	NA	NA	0.9165	1.0213	1.0527	0.8562	0.4446	0.5467
18	B	0.3158	-0.1079	-1.0174	-1.4023	NA	NA	-1.5555	-1.7732	-1.7501	-1.8100	0.6112	0.6805
19	B	-0.3796	-0.9207	2.1503	1.8606	NA	NA	-0.2506	-0.2548	-1.0279	-0.8536	0.8178	0.7486
20	W	NA	NA	-0.5118	-0.4171	-0.3109	0.2335	0.9822	0.5202	0.2268	-0.0132	0.7244	0.7369
21	B	0.2354	-0.4416	1.0415	0.3766	NA	NA	-0.1370	-0.1748	0.6289	0.5646	0.0659	0.0414
22	B	NA	NA	-0.7704	-0.7109	-1.2318	-1.0596	-1.8894	-1.8348	1.4781	0.7385	-0.1251	-0.2995

Standardized, adjusted PRS ($PRS_{std-adj}$) are shown for each of 6 diseases and 22 samples with both whole genome sequencing (WGS) and imputed genotyping arrays (IMPU). Green indicates a concordant categorization above the high-risk threshold from both WGS and IMPU for that sample and disease. Yellow indicates a discordant categorization around the high-risk threshold for the sample and disease. All other cells indicate PRS with concordant categorization below the high-risk threshold for that sample and disease. NA indicates cells where the PRS was not calculated for that disease-sex combination. Abbreviations: AFib, atrial fibrillation; B, reported Black race; BrCa, breast cancer; CAD, coronary artery disease; CRCa, colorectal cancer; PrCa, prostate cancer; T2D, type 2 diabetes mellitus; U, unknown race; W, reported white race.

Supplemental Table 9. Comparison between standardized, adjusted PRS scores from MGBB data and prospective assay for diseases in nine samples with high risk

Sample	Disease	MEG	GDA
1	CRCa	1.9337	1.8773
2	CAD	2.1961	2.1347
3	PrCa	1.0736	1.1675
4	CRCa	2.7145	2.7161
5	AF	1.6851	1.6435
6	T2D	2.4144	2.2023
7	CAD	4.0646	4.0855
8	PrCa	4.1363	4.1380
9	BrCa	4.0142	4.4274
	CAD	1.7722	1.8204

Shown are 9 MGBB samples determined to be at high risk for a total of 10 diseases using MEG array data. Standardized, adjusted PRS ($PRS_{std\text{-}adj}$) are shown for both the MGBB data and for the same samples run using the GDA array in the prospective assay. Abbreviations: AFib, atrial fibrillation; BrCa, breast cancer; CAD, coronary artery disease; CRCa, colorectal cancer; GDA, Global Diversity Array; MEG, Multi-Ethnic Global array; PrCa, prostate cancer; PRS, polygenic risk score; T2D, type 2 diabetes mellitus.

PARTNERS HEALTHCARE | PERSONALIZED MEDICINE
MASSACHUSETTS GENERAL HOSPITAL
BRIGHAM AND WOMEN'S HOSPITAL

LABORATORY FOR MOLECULAR MEDICINE
65 Landsdowne St, Cambridge, MA 02139
Phone: (617) 768-8500 / Fax: (617) 768-8513
http://www.partners.org/personalizedmedicine/lmm

Name: **LAST, FIRST**	MRN: **XXXXX**	LMM Accession ID: **PM-19-X00000**
DOB: **MM/DD/XXXX**	Referring Facility: **XXXX**	Specimen: **Blood, peripheral**
Sex: **Female/Male**	Referring Physician: **XXXX**	Received: **MM/DD/XXXX**
Family #: **F00000**		Page: **1 of X**
Test Performed: **GenoVA Polygenic Risk Assessment**	Test Codes:	

RESULTS SUMMARY*

HIGH POLYGENIC DISEASE RISK: Genotyping indicated an increased polygenic risk for developing colorectal cancer. Result details are provided below.

***Polygenic risk calculated using data from predominantly European ancestry individuals. Results are known to be less accurate for non-European ancestry individuals. See details below.**

DETAILED GENOMIC RESULTS

A. POLYGENIC DISEASE RISK

Polygenic risk describes the chance of developing certain health conditions based on a large number of genetic variants across the genome. This test assessed the risk for developing the following conditions: atrial fibrillation, coronary artery disease, type 2 diabetes, colorectal cancer, and prostate cancer (for patients with a prostate) or breast cancer (for patients born with female breast tissue).

This test identified an **increased polygenic risk for colorectal cancer** (see methodology for complete description of the analysis). It **did NOT** indicate increased polygenic risk for the remaining conditions.

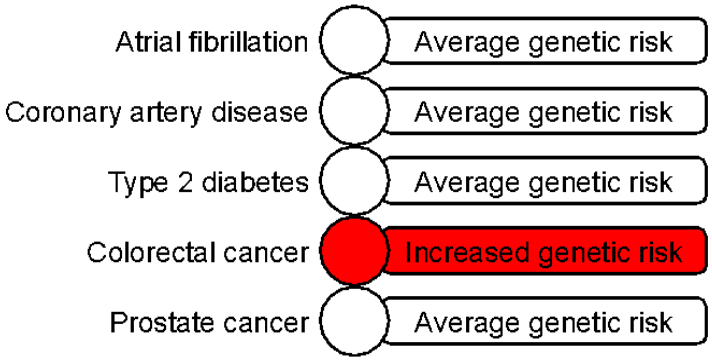

Diseases WITH an increased polygenic risk

Disease	This patient's result	General disease prevalence
Colorectal cancer	Increased polygenic risk	1 in 24 people
**RISK INTERPRETATION:** The patient's calculated polygenic risk score, derived from 80 loci, has been associated with an INCREASED risk for colorectal cancer, defined here as greater than 2-fold risk. Individuals with similar polygenic risk scores have been shown to have an increased risk for colorectal cancer above baseline. Values of this polygenic risk score that fall among the top 5% were associated with a greater than 2-fold greater risk of developing colorectal cancer among >125,000 individuals of European ancestry when compared to the average individual (Huyghe 2019); similar association was observed among 21,630 individuals of East Asian ancestry (Schmit 2018).		
**DISEASE INFORMATION:** Colorectal cancer is uncontrolled growth in the colon or rectum. It begins as polyps that may turn into cancer over time. Colorectal cancer is the 3rd most common cancer in the United States, and is the 2nd leading cause of cancer death. Colorectal cancer is most often found in people 50 years or older. Symptoms include blood in the stool, stomach pain, aches or cramps, and unintentional weight loss, but many patients have no symptoms (adapted from Centers for Disease Control and Prevention https://www.cdc.gov/cancer/colorectal/, SEER https://seer.cancer.gov/statfacts/html/colorect.html and Colorectal Cancer Alliance https://www.ccalliance.org/colorectal-cancer-information/what-is-colorectal-cancer).		

LABORATORY FOR MOLECULAR MEDICINE
65 Landsdowne St, Cambridge, MA 02139
Phone: (617) 768-8500 / Fax: (617) 768-8513
http://www.partners.org/personalizedmedicine/lmm

Diseases WITHOUT an increased polygenic risk

Disease	This patient's result	General disease prevalence
Atrial fibrillation	Average polygenic risk	Lifetime risk of 1 in 3 to 1 in 5

RISK INTERPRETATION: The patient's calculated polygenic risk score, derived from 6730143 loci, has NOT been associated with high risk for atrial fibrillation, defined here as greater than 2-fold risk. For the majority of individuals with polygenic risk scores similar to this, risk for atrial fibrillation is not likely to be increased above baseline. On the other hand, values of this polygenic risk score that fall among the top 7% were associated with a greater than 2-fold greater risk of developing atrial fibrillation among >400,000 British volunteers of European ancestry when compared to the average individual (Khera 2018).

DISEASE INFORMATION: Atrial fibrillation (AFib) is an inherited abnormality of the heart's normal rhythm due to episodes of uncoordinated electrical activity in the heart's upper chambers. Symptoms include dizziness, chest pain, sensations of fluttering or chest palpitations, shortness of breath and fainting. AFib and complications associated with the condition (such as stroke and heart failure) may occur at any age, but risk of developing symptoms increases with age (adapted from Genetics Home Reference https://ghr.nlm.nih.gov/condition/familial-atrial-fibrillation and Centers for Disease Control and Prevention https://www.cdc.gov/heartdisease/atrial_fibrillation.htm).

Disease	This patient's result	General disease prevalence
Coronary artery disease	Average polygenic risk	1 in 5 men aged 60-79<br>1 in 8 women aged 60-79

RISK INTERPRETATION: The patient's calculated polygenic risk score, derived from 6630016 loci, has NOT been associated with high risk for coronary artery disease, defined here as greater than 2-fold risk. For the majority of individuals with polygenic risk scores similar to this, risk for coronary artery disease is not likely to be increased above baseline. On the other hand, values of this polygenic risk score that fall among the top 10% were associated with a greater than 2-fold greater risk of developing coronary artery disease among >400,000 British volunteers of European ancestry when compared to the average individual (Khera 2018). Having an ancestry-adjusted score in the top 5th percentile has also been associated with an odds ratio of early myocardial infarction (before age 55) of 5.09, 2.02, 3.38, and 3.33 in people of white, black, Hispanic, and Asian ancestry, respectively (Khera 2019).

DISEASE INFORMATION: Coronary artery disease (CAD) is the most common type of heart disease in the United States, caused by plaque buildup in the walls of the coronary arteries, which supply blood to the heart. Risk of developing CAD increases with age. Symptoms of CAD include chest pain (angina), weakness, light-headedness, nausea, pain or discomfort in the arms or shoulder, shortness of breath, and heart attack (adapted from Centers for Disease Control and Prevention https://www.cdc.gov/heartdisease/coronary_ad.htm).

Disease	This patient's result	General disease prevalence
Prostate cancer	Average polygenic risk	1 in 8 men

RISK INTERPRETATION: The patient's calculated polygenic risk score, derived from 137 loci, has NOT been associated with high risk for prostate cancer, defined here as greater than 2-fold risk. For the majority of individuals with polygenic risk scores similar to this, risk for prostate cancer is not likely to be increased above baseline. On the other hand, values of this polygenic risk score that fall among the top 13% were associated with a greater than 2-fold greater risk of developing prostate cancer among >140,000 men of European ancestry when compared to the average individual (Schumacher 2018).

DISEASE INFORMATION: Prostate cancer is characterized by abnormal cell growth in the prostate. All men are at risk for prostate cancer; however, the risks for developing and dying from prostate cancer increase with age, with the highest incidence being observed in men ≥ 65. Prevalence of prostate cancer varies among different ethnic and racial groups, with the highest prevalence observed in African-American men. Prostate cancer is often asymptomatic in its early stages but can be associated with bone pain if it spreads to other parts of the body (adapted from Centers for Disease Control and Prevention www.cdc.gov/cancer/prostate/basic_info/index.htm and www.ncbi.nlm.nih.gov/pmc/articles/PMC6497009/).

Disease	This patient's result	General disease prevalence
Type 2 diabetes	Average polygenic risk	More than 1 in 5 people >65 years old

RISK INTERPRETATION: The patient's calculated polygenic risk score, derived from 6917079 loci, has NOT been associated with high risk for type 2 diabetes, defined here as greater than 2-fold risk. For the majority of individuals with polygenic risk scores similar to this, risk for type 2 diabetes is not likely to be increased above baseline. On the other hand, values of this polygenic risk score that fall among the top 8% were associated with a greater than 2-fold greater risk of developing type 2 diabetes among >400,000 British volunteers of European ancestry when compared to the average individual (Khera 2018).

DISEASE INFORMATION: Type 2 diabetes (T2D) is characterized by high blood sugar levels due to abnormal insulin processing. T2D typically occurs during middle or late adulthood and progresses over time. Symptoms include polyuria, polydipsia, fatigue, blurred vision, neuropathy, and weight loss (adapted from Genetics Home Reference https://ghr.nlm.nih.gov/condition/type-2-diabetes).

[FOR FEMALES]

Disease	This patient's result	General disease prevalence

Breast cancer	Average polygenic risk	1 in 8 women
**RISK INTERPRETATION:** The patient's calculated polygenic risk score, derived from 3820 loci, has NOT been associated with high risk for breast cancer, defined here as greater than 2-fold risk. For the majority of individuals with polygenic risk scores similar to this, risk for breast cancer is not likely to be increased above baseline. On the other hand, values of this polygenic risk score that fall among the top 8% were associated with a greater than 2-fold greater risk of developing breast cancer among >380,000 women of European ancestry when compared to the average individual (Mavaddat 2019).		
**DISEASE INFORMATION:** Breast cancer is a disease characterized by the multiplication of abnormal breast cells to form a tumor. Breast cancer can develop in both men and women, but is much more common in females. Breast cancer is the second most common cancer in women, and about 1 in 8 women in the United States will develop breast cancer during her lifetime. Symptoms include a lump or thickening in or near the breast, changes in breast size or shape, nipple discharge, tenderness, inversion of nipples, and skin irritation, dimpling, or scaliness. Many women with breast cancer have no symptoms early in the disease (adapted from Genetic Home Reference https://ghr.nlm.nih.gov/condition/breast-cancer).		

Limitations: The summary risk assessments above are based on combining individual risk allele data in ways that may not always apply to each individual patient. These results should be interpreted in the context of the patient's medical evaluation, family history, and racial/ethnic background. The performance of polygenic risk scores is typically more robust across European ancestries than non-European ancestries.

B. ACTIONABLE MONOGENIC DISEASE FINDINGS

This genotyping test did NOT identify any of the tested medically significant variants in a list of 59 actionable genes (See methodology). Pathogenic variants in these genes are associated with an increased risk of developing certain conditions, including some cancers, heart diseases, and other disorders. NOTE, this assay did not interrogate all possible pathogenic variation within these 59 genes, and thus a negative result does not reflect an absence of a disease-causing variant in one or more of these genes in this individual.

Limitations: As a genotyping assay, this test cannot detect variants which are not specifically targeted on the array and thus should not be considered a screening assay for these genes. Specific types of genetic variation, such as triplet repeat expansions, structural variation, and copy number events, or sequence variants not included in the list of genotyped variants are currently not reliably detected by this assay. Variant interpretation may change over time if more information becomes available. Please note that the presence of pathogenic variants in variants not part of the assay, genes not analyzed, or regions not captured by filtering strategies cannot be fully excluded.

RECOMMENDATIONS

These results should be interpreted in the context of this individual's personal medical history and family history. [IF ACTIONABLE MONOGENIC DISEASE FINDING: Genetic counseling is recommended for this individual and their relatives. Familial variant testing for the *GENE* variant is available if desired.]

METHODOLOGY

Genotyping:

Genomic DNA was genotyped using the Illumina Global Diversity Array and iScan instrumentation by the Clinical Research Sequencing Platform of the Broad Institute. Illumina's Autocall genotype calling software (IAAP) determines genotyping results in GTC format from image data scanned on the Illumina genotyping array. Samples were required to meet or exceed the 98% call rate specification by Autocall in order to be considered passing. The GTC file generated by Autocall is converted to a VCF file using internally validated software. The VCF file from the GDA is imputed using 1000 Genomes as the reference population to determine the likely allele state at sites across the genome using an imputation pipeline that includes quality control, phasing (using EAGLE) and imputation (using minimac4).

Polygenic Disease Risk:

This test assessed the polygenic risk for developing the following six conditions as previously identified: breast cancer (in females), prostate cancer (in males), colorectal cancer, atrial fibrillation, coronary artery disease, and type 2 diabetes (Huyghe et al., 2019; Khera et al., 2018; Mavaddat., 2018; Schumacher et al., 2018). For each condition, a population-standardized PRS is computed as the sum of the patient's risk alleles across multiple SNPs weighted by the SNP-specific effects reported in large genome-wide association studies. An individual is considered to be at an increased PRS if their standardized PRS is above the threshold score for an OR>2 as compared to the median PRS value [computed as ln(2)/ln(change in OR per SD change in PRS)].

Actionable Monogenic Disease Findings:

The American College of Medical Genetics and Genomics (ACMG) recommends reporting variants discovered in certain genes when discovered as secondary findings in a genomics assay. These variants cause conditions that are considered actionable, meaning there are specific guidelines available to monitor and/or treat these conditions. If identified, only those variants recommended by ACMG are reported. Note, this test will miss pathogenic or likely pathogenic variants not included in the genotyping array. Variants present on the array in these 59 genes have been filtered to include: (1) variants identified by our laboratory to be pathogenic or likely pathogenic; (2) variants classified as disease causing mutations in public

databases that have a minor allele frequency <5.0% in the Genome Aggregation Database (gnomAD, http://gnomadexac.broadinstitute.org/); and (3) nonsense, frameshift, and +/-1,2 splice-site variants in disease-associated genes with a minor allele frequency ≤1.0% in gnomAD. The evidence for phenotype-causality has been evaluated for each variant identified from the filtering strategies listed above and variants are classified based on ACMG/AMP criteria (Richards et al. 2015) with ClinGen rule specifications (https://www.clinicalgenome.org/working-groups/sequence-variant-interpretation/). Variants are reported according to HGVS nomenclature (http://varnomen.hgvs.org/). Only those variants with evidence for causing or contributing to disease are reported. All disease-associated variants on this report are confirmed via Sanger sequencing or another orthogonal technology. Please contact the laboratory for additional information or for a complete list of variants and genes analyzed.

The initial genotyping component of this test was performed by the Clinical Research Sequencing Platform of the Broad Institute (320 Charles St, Cambridge, MA 02141; CLIA#22D2055652), and the Sanger confirmation, interpretive algorithms and clinical reports were generated by the Laboratory for Molecular Medicine at Partners Healthcare Personalized Medicine (LMM, 65 Landsdowne St, Cambridge, MA 02139; 617-768-8500; CLIA#22D1005307). This test has not been cleared or approved by the U.S. Food and Drug Administration (FDA). The FDA has determined that such clearance or approval is not necessary.

REFERENCES

Huyghe, J. R., Bien, S. A., Harrison, T. A., Kang, H. M., Chen, S., Schmit, S. L., . . . Peters, U. (2019). Discovery of common and rare genetic risk variants for colorectal cancer. *Nat Genet, 51*(1), 76-87. doi:10.1038/s41588-018-0286-6

Kalia, S. S., Adelman, K., Bale, S. J., Chung, W. K., Eng, C., Evans, J. P., . . . Miller, D. T. (2017). Recommendations for reporting of secondary findings in clinical exome and genome sequencing, 2016 update (ACMG SF v2.0): a policy statement of the American College of Medical Genetics and Genomics. *Genet Med, 19*(2), 249-255. doi:10.1038/gim.2016.190

Khera, A. V., Chaffin, M., Aragam, K. G., Haas, M. E., Roselli, C., Choi, S. H., . . . Kathiresan, S. (2018). Genome-wide polygenic scores for common diseases identify individuals with risk equivalent to monogenic mutations. *Nat Genet, 50*(9), 1219-1224. doi:10.1038/s41588-018-0183-z

Mavaddat, N., Michailidou, K., Dennis, J., Lush, M., Fachal, L., Lee, A., . . . Easton, D. F. (2019). Polygenic Risk Scores for Prediction of Breast Cancer and Breast Cancer Subtypes. *Am J Hum Genet, 104*(1), 21-34. doi:10.1016/j.ajhg.2018.11.002

Schumacher, F. R., Al Olama, A. A., Berndt, S. I., Benlloch, S., Ahmed, M., Saunders, E. J., . . . Mechanisms in Oncology /Elucidating Loci Involved in Prostate Cancer Susceptibility, C. (2018). Association analyses of more than 140,000 men identify 63 new prostate cancer susceptibility loci. *Nat Genet, 50*(7), 928-936. doi:10.1038/s41588-018-0142-8

Report Preparation by:
Final Report by:

U.S. Department of Veterans Affairs
Veterans Health Administration
Office of Research & Development

GenoVA
Genomic Medicine at VA

High Genetic Risk: Atrial Fibrillation

Based on analysis of your genetic sample, you have high genetic risk of developing atrial fibrillation (AFib) at some point in your life. These results do not indicate that you have atrial fibrillation now or will definitely develop atrial fibrillation in the future. There are steps you and your healthcare team can take to prevent development of the disease or diagnose and treat it early.

It is recommended that you consult with your healthcare provider and develop a plan to reduce your risk.

What Is Atrial Fibrillation (AFib)?

Atrial fibrillation (AFib) is the most common form of heart arrhythmia. An arrhythmia is when the heart beats in an irregular way. AFib causes irregular beating and abnormal blood flow in the heart, which can lead to blood clots. AFib can occur in brief episodes or it can be a chronic condition.[1]

Who Gets Atrial Fibrillation?

Both men and women can get AFib. Risk of developing AFib increases with age.

What Are Risk Factors for AFib?

In addition to your genetic results indicating high AFib risk, your risk may be higher than average if:

- You have heart disease or high blood pressure.
- You have hyperthyroidism (overactive thyroid).
- You consume alcohol.[2]

What Are the Signs and Symptoms?

AFib may be associated with symptoms such as irregular heartbeat, palpitations, weakness, and shortness of breath. Some people have no symptoms from AFib.[3]

What Can I Do To Reduce My Risk?

You can reduce your risk of getting AFib by:

- Limiting your alcohol consumption.
- Increasing your physical activity.
- Maintaining a healthy weight.
- Increasing your intake of healthy fats such as fish oil and extra virgin olive oil.

How Can I Find Out If I Have AFib?

Your healthcare provider might suspect you have atrial fibrillation based on an irregular pulse or heartbeat. This can be confirmed with an electrocardiogram (ECG) or other types of heart monitoring.

---

[1] Centers for Disease Control and Prevention. Atrial Fibrillation Fact Sheet. 2019; https://www.cdc.gov/dhdsp/data_statistics/fact_sheets/fs_atrial_fibrillation.htm

[2] Larsson SC, Drca N, Wolk A. Alcohol consumption and risk of atrial fibrillation: a prospective study and dose-response meta-analysis. *Journal of the American College of Cardiology.* 2014;64(3):281-289.

[3] January CT, Wann LS, Alpert JS, et al. 2014 AHA/ACC/HRS guideline for the management of patients with atrial fibrillation: executive summary: a report of the American College of Cardiology/American Heart Association Task Force on practice guidelines and the Heart Rhythm Society. *Circulation.* 2014;130(23):2071-2104.

VA | U.S. Department of Veterans Affairs
Veterans Health Administration
Office of Research & Development

GenoVA
Genomic Medicine at VA

High Genetic Risk: Breast Cancer

Based on analysis of your genetic sample, you have high genetic risk of developing breast cancer at some point in your life. These results do not indicate that you have or will definitely develop breast cancer in the future. There are steps you and your healthcare team can take to prevent development of the disease or diagnose and treat it early.

It is recommended that you consult with your healthcare provider and develop a plan to reduce your risk.

What Is Breast Cancer?

Breast cancer is among the top two most common cancers found among women, second only to skin cancer.[1] Breast cancer is a disease in which cells in the breast grow out of control.[2]

Who Gets Breast Cancer?

Both women and men can develop breast cancer. Risk of breast cancer increases with age.

What Are Risk Factors for Breast Cancer?

In addition to the status of your genetic results, your risk for breast cancer may be higher than average if:

- You consume alcohol.[3,4,5]
- You smoke.[1,2,3]
- You have a higher than normal body-mass index (BMI).
- You started menstruating before age 12.[6]
- You started menopause after age 55.[6]
- You have a family history of breast cancer (parent, sibling, or child).[6]
- You have never given birth or were older when your first child was born.[6]
- You have or are taking hormones to replace missing estrogen and progesterone in menopause for more than five years.[6]

What Are the Signs and Symptoms?

Breast cancer may be associated with symptoms such as breast masses, overlying skin changes, or nipple discharge.

What Can I Do To Reduce My Risk?

You can reduce your risk of getting breast cancer by:

- Increasing your physical activity.
- Maintaining a healthy diet (low sodium, low fat).
- Maintaining a normal blood pressure reading (average blood pressure below 130/80).

How Can I Find Out If I Have Breast Cancer?

Talk with your healthcare provider about breast cancer screening. Most women who are aged 50-74 years old should have a screening mammogram (X-ray of the breast) every two years.

[1] Mayo Clinic. Breast Cancer: Symptoms & Causes. 2019; https://www.mayoclinic.org/diseases-conditions/breast-cancer/symptoms-causes/syc-20352470
[2] Centers for Disease Control and Prevention. Breast Cancer: What are the symptoms of Breast Cancer?.2019; https://www.cdc.gov/cancer/breast/basic_info/symptoms.htm
[3] White AJ, DeRoo LA, Weinberg CR, Sandler DP. Lifetime Alcohol Intake, Binge Drinking Behaviors, and Breast Cancer Risk. *American Journal of Epidemiology.* 2017;186(5):541-549.
[4] Gram IT, Park SY, Kolonel LN, et al. Smoking and Risk of Breast Cancer in a Racially/Ethnically Diverse Population of Mainly Women Who Do Not Drink Alcohol: The MEC Study. *American Journal of Epidemiology.* 2015;182(11):917-925.
[5] Miller MD, Marty MA, Broadwin R, et al. The association between exposure to environmental tobacco smoke and breast cancer: a review by the California Environmental Protection Agency. *Prev Med.* 2007;44(2):93-106.
[6] Centers for Disease Control and Prevention. Breast Cancer: What You Need to Know. 2019; https://www.cdc.gov/cancer/breast/pdf/breastcancerfactsheet.pdf

High Genetic Risk: Colorectal Cancer

Based on analysis of your genetic sample, you have high genetic risk of developing colorectal cancer (CRC) at some point in your life. These results do not indicate that you have CRC now or will definitely develop CRC in the future. There are steps you and your healthcare team can take to prevent development of the disease or diagnose and treat it early.

It is recommended that you consult with your healthcare provider and develop a plan to reduce your risk.

What Is Colorectal Cancer?

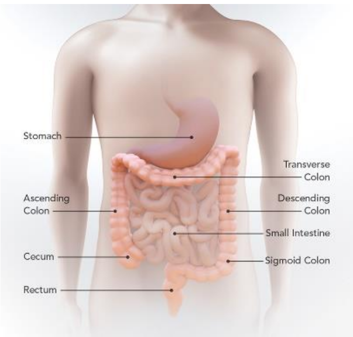

Colorectal cancer is cancer that occurs in the colon (large intestine) or rectum (the passageway that connects the colon to the anus). Abnormal growths, called polyps, can sometimes form in the colon or rectum and, over time, can turn into CRC.[1] CRC is the fourth most common cancer diagnosed among adults and the second leading cause of death from cancer in the US.[2]

Who Gets Colorectal Cancer?

Both men and women can get CRC. It is most often found in people aged 50 or older, and the risk increases with age.

What Are Risk Factors for CRC?

In addition to your genetic results indicating high CRC risk, your risk may be higher than average if:

- You smoke and/or consume processed and/or red meats, such as deli meats (i.e. bologna, ham, salami, etc.), sausage, bacon, and beef.[3]
- You or a close relative have had colorectal polyps or colorectal cancer.[4]
- You have inflammatory bowel disease, Crohn's disease, or ulcerative colitis.[6]
- You have a genetic syndrome such as familial adenomatous polyposis (FAP) or hereditary nonpolyposis colorectal cancer.[6]

What Are the Signs and Symptoms?

CRC may be associated with change in bowel habits or rectal bleeding.[2,5] However, it is important to note that CRC can start with no symptoms. This means that someone could have CRC and not know it. That is why having a screening test is so important.

What Can I Do To Reduce My Risk?

You can reduce your risk of getting CRC by:

- Asking your doctor about getting a CRC screening test, such as a colonoscopy or fecal occult blood testing.
- Getting and staying active.
- Reducing or eliminating red and processed meats from your diet.
- Maintaining a healthy weight.
- Quitting smoking.

[1] Centers for Disease Control and Prevention. What is Colorectal Cancer?. 2019; https://www.cdc.gov/cancer/colorectal/basic_info/what-is-colorectal-cancer.htm
[2] Wolf AMD, Fontham ETH, Church TR, et al. Colorectal cancer screening for average-risk adults: 2018 guideline update from the American Cancer Society. *CA: A Cancer Journal for Clinicians.* 2018;68(4):250-281.
[3] Bouvard V, Loomis D, Guyton KZ, et al. Carcinogenicity of consumption of red and processed meat. The Lancet. Oncology. 2015 Dec;16(16):1599-1600. DOI: 10.1016/s1470-2045(15)00444-1.
[4] https://www.cdc.gov/cancer/colorectal/pdf/Basic_FS_Eng_Color.pdf
[5] Provenzale, D., Gupta, S., Ahnen, D. J., et al. (2018). NCCN Guidelines Insights: Colorectal Cancer Screening, Version 1.2018, *Journal of the National Comprehensive Cancer Network J Natl Compr Canc Netw*, *16*(8), 939-949.

U.S. Department of Veterans Affairs
Veterans Health Administration
Office of Research & Development

GenoVA
Genomic Medicine at VA

High Genetic Risk: Coronary Artery Disease

Based on analysis of your genetic sample, you have high genetic risk of developing coronary artery disease (CAD) at some point in your life. These results do not indicate that you have CAD now or will definitely develop it in the future. There are steps you and your healthcare team can take to prevent development of the disease or diagnose and treat it early.

It is recommended that you consult with your healthcare provider and develop a plan to reduce your risk.

What Is Coronary Artery Disease?

Coronary artery disease (CAD) is the most common form of heart disease in the US. CAD is caused by plaque buildup in the wall of the coronary arteries (arteries that supply blood to the heart).[1] Over time, plaque buildup can cause the inside of the arteries to narrow, which could partially or totally block blood flow. When your heart muscle doesn't get enough blood, you may experience chest pain (angina), a heart attack (myocardial infarction), or even sudden death.

Who Gets Coronary Artery Disease?

Both men and women can get CAD. Risk increases with age.

What Are Risk Factors for CAD?

In addition to your genetic results indicating high risk for CAD, your risk for CAD may be higher than average if:

- You are overweight or obese.[2,3]
- You have high blood pressure.
- You have high levels of "bad" cholesterol (LDL).
- You smoke.

What Are the Signs and Symptoms?

CAD may be associated with symptoms such as pain in the chest or shortness of breath.

What Can I Do To Reduce My Risk?

You can reduce your risk of getting CAD by:

- Increasing your physical activity to 150 minutes of moderate-intensity aerobic physical activity or 75 minutes of vigorous aerobic physical activity per week.
- Maintaining a Mediterranean-style diet (no added sodium, low saturated fat, high polyunsaturated fat, low refined carbohydrates, high fiber).
- Maintaining a normal blood pressure reading (average blood pressure below 130/80).[4]
- Talking to your doctor about taking medications to treat risk factors, such as statins or aspirin.

People at high genetic risk for CAD may be able to reduce their risk of heart attack by up to 50%, with a healthy lifestyle including diet, exercise, maintaining a normal weight, and not smoking.[5]

How Can I Find Out If I May Have CAD?

Your healthcare provider might suspect you have CAD based on symptoms such as chest pain or shortness of breath, or based on abnormalities on an electrocardiogram (ECG). They might order a stress test, a coronary CT scan, or other tests to see whether there is any blockage in the coronary arteries.

[1] Centers for Disease Control and Prevention. Coronary Artery Disease. 2019; https://www.cdc.gov/heartdisease/coronary_ad.htm
[2] Stone NJ, Robinson J, Lichtenstein AH, et al. 2013 ACC/AHA Guideline on the Treatment of Blood Cholesterol to Reduce Atherosclerotic Cardiovascular Risk in Adults: A Report of the American College of Cardiology/American Heart Association Task Force on Practice Guidelines. *Circulation.* 2014;129(25 Suppl 2):S1-45.
[3] Gibbons RJ, Balady GJ, Bricker JT, et al. ACC/AHA 2002 guideline update for exercise testing: summary article. A report of the American College of Cardiology/American Heart Association Task Force on Practice Guidelines (Committee to Update the 1997 Exercise Testing Guidelines). *Journal of the American College of Cardiology.* 2002;40(8):1531-1540.
[4] Whelton PK, Carey RM, Aronow WS, et al. 2017 ACC/AHA/AAPA/ABC/ACPM/AGS/APhA/ASH/ASPC/NMA/PCNA Guideline for the Prevention, Detection, Evaluation, and Management of High Blood Pressure in Adults: Executive Summary: A Report of the American College of Cardiology/American Heart Association Task Force on Clinical Practice Guidelines. Hypertension. 2018 Jun;71(6):1269-1324.
[5] Khera AV, Emdin CA, Drake I, et al. Genetic Risk, Adherence to a Healthy Lifestyle, and Coronary Disease. *NEJM* 2016; 375:2349-2358.

High Genetic Risk: Prostate Cancer

Based on analysis of your genetic sample, you have high genetic risk of developing prostate cancer at some point in your life. These results do not indicate that you have prostate cancer now or will definitely develop prostate cancer in the future. There are steps you and your healthcare team can take to prevent development of the disease or diagnose and treat it early.

It is recommended that you consult with your healthcare provider and develop a plan to reduce your risk.

What Is Prostate Cancer?

Prostate cancer is among the top two most common cancers in men, second only to skin cancer. Prostate cancer is a disease in which cells in the prostate grow out of control.[1]

Who Gets Prostate Cancer?

All men are at risk for prostate cancer. The most common risk factor is age – the older a man gets, the greater the chance of getting prostate cancer.[2] African-Americans are more likely to get prostate cancer than other race/ethnic groups.

What Are Risk Factors for Prostate Cancer?

In addition to your genetic results indicating high prostate cancer risk, your risk may be higher than average if:

- You smoke.[3]
- You were exposed to Agent Orange.
- You eat a diet high in animal fat and low in vegetables.

What Are the Signs and Symptoms?

Prostate cancer most often has no symptoms. If it becomes advanced, it can be associated with symptoms such as bone pain. Your provider might identify prostate nodules or asymmetry during your physical examination.

What Can I Do To Reduce My Risk?

You can reduce your risk of getting prostate cancer by:

- Maintaining a healthy weight.
- Quitting smoking.

How Can I Find Out If I May Have Prostate Cancer?

Ask your doctor about screening for prostate cancer. They might recommend getting a prostate specific antigen (PSA) test,[4] a commonly used blood test to screen for prostate cancer.

PSA is a substance created by the prostate. PSA levels in the blood can be higher in men who have prostate cancer, though PSA levels can also be affected by certain medical procedures, certain medications, an enlarged prostate, or a prostate infection. Your healthcare provider is the best person to interpret your PSA test results, as many factors can affect PSA levels.

If your PSA level is abnormally high, your provider might recommend additional testing, such as a prostate biopsy, to look at prostate tissue under a microscope for evidence of cancer.

[1] Centers for Disease Control and Prevention. What is Prostate Cancer?. 2019; https://www.cdc.gov/cancer/prostate/basic_info/what-is-prostate-cancer.htm
[2] Centers for Disease Control and Prevention. Who Is at Risk for Prostate Cancer?. 2019; https://www.cdc.gov/cancer/prostate/basic_info/risk_factors.htm
[3] Islami F, Moreira DM, Boffetta P, Freedland SJ. A systematic review and meta-analysis of tobacco use and prostate cancer mortality and incidence in prospective cohort studies. *European Urology.* 2014;66(6):1054-1064.
[4] Grossman DC, Curry SJ, Owens DK, et al. Screening for Prostate Cancer: US Preventive Services Task Force Recommendation Statement. *JAMA.* 2018;319(18):1901-1913.

High Genetic Risk: Type 2 Diabetes

Based on analysis of your genetic sample, you have high genetic risk of developing type 2 diabetes mellitus (T2D) at some point in your life. These results do not indicate that you have T2D now or will definitely develop T2D in the future. There are steps you and your healthcare team can take to prevent development of the disease or diagnose and treat it early.

It is recommended that you consult with your healthcare provider and develop a plan to reduce your risk.

What Is Type 2 Diabetes (T2D)?

Type 2 diabetes (T2D) is a chronic condition that affects the way your body breaks down sugar (glucose). When you have T2D, one of two things may occur: (1) your body has trouble regulating insulin (a hormone that helps to regulate the movement of sugar in your cells)[1]; or (2) your body doesn't produce enough insulin to maintain normal glucose levels. Over time, untreated high glucose levels can damage organs such as heart, kidneys, and eyes.

Who Gets T2D?

Both men and women can get T2D, and risk increases with age.[2]

What Are Risk Factors for T2D?

In addition to your genetic results indicating high T2D risk, your risk may be higher than average if:

- You are overweight or obese.
- You engage in little or no physical activity.
- You have a family history of diabetes
- You are of a non-white race/ethnic group.
- You smoke.
- You have a high consumption of red meat, processed meat, high fat dairy products, sweets, and desserts.

What Are the Signs and Symptoms?

T2D often has no symptoms at first. It can be associated with symptoms such as frequent urination (polyuria), excessive thirst (polydipsia), and numbness or tingling in the hands or feet (neuropathy).

What Can I Do To Reduce My Risk?

You can reduce your risk of getting type 2 diabetes by:

- Increasing your physical activity.
- Maintaining a healthy weight.
- Eating a Mediterranean-style diet high in fruits, vegetables, nuts, whole grains, and olive oil.

How Can I Find Out If I May Have T2D?

Ask your doctor about getting a blood test to screen for diabetes. Such blood tests include a fasting glucose test, a hemoglobin A1c (HbA1c) test, or 2-hour oral glucose tolerance test.

If you are found to have a condition called prediabetes, your provider might recommend that you lose weight or start a medication to prevent the development of T2D.

[1] Mayo Clinic Type 2 Diabetes: overview. 2019; https://www.mayoclinic.org/diseases-conditions/type-2-diabetes/symptoms-causes/syc-20351193

[2] Centers for Disease Control and Prevention. Type 2 Diabetes. 2019; https://www.cdc.gov/diabetes/basics/type2.html

High Genetic Risk: Atrial Fibrillation

Your patient's genetic analysis indicates that he/she is at HIGH GENETIC RISK for atrial fibrillation (top 7th percentile of genetic risk). This means that he/she has a greater than 2.0-fold higher risk of developing AFib compared to a person with an average genetic risk score.

The genetic score was calculated from millions of genetic markers in your patient's DNA. In prior studies, this score was found to be highly correlated with risk of AFib.[1] This genetic test does not account for other disease risk factors, such as age, alcohol intake, or cardiovascular disease.

Currently, there are no professional guidelines on how to manage a patient with high genetic risk for AFib or whether that management should differ from the average population. This letter provides you with general recommendations for screening for and diagnosing AFib and helping your patients lower their risk for AFib.

Signs and Symptoms

Consider asking your patient about symptoms such as palpitations, weakness, and dyspnea. Look for an irregular heart rhythm on pulse or auscultation.[2]

Screening and Diagnosis

There are currently no professional guidelines on whether screening recommendations should differ for patients with high genetic risk for AFib compared with the average population.

If AFib is suspected, consider diagnostic testing with office or ambulatory electrocardiogram (ECG) monitoring.

Newer technologies such as smart watches with ECG capabilities currently have an uncertain role in clinical care.

Risk Factor Optimization

In addition to genetic predisposition for developing AFib, there are other factors that may raise or lower your patient's risk:

- Hypertensive heart disease: Patients should keep their blood pressure below 130/80.[3]
- Alcohol consumption: Limiting alcohol intake is associated with lower AFib incidence across all categories of genetic risk scores.[4]
- Obesity: Long-term weight reduction might limit arrhythmia burden in patients with atrial fibrillation.[5] Observational studies suggest that maintaining a BMI<25 kg/m^2 might have even greater benefit in preventing AFib in patients with a high-risk genetic score.[6]

[1] Khera AV, Chaffin M, Aragam KG, et al. Genome-wide polygenic scores for common diseases identify individuals with risk equivalent to monogenic mutations. *Nature Genetics.* 2018;50(9):1219-1224.
[2] January CT, Wann LS, Alpert JS, et al. 2014 AHA/ACC/HRS guideline for the management of patients with atrial fibrillation: executive summary: a report of the American College of Cardiology/American Heart Association Task Force on practice guidelines and the Heart Rhythm Society. *Circulation*. 2014; 130(23):2071-2104
[3] Whelton PK, Carey RM, Aronow WS, et al. 2017 ACC/AHA/AAPA/ABC/ACPM/AGS/APhA/ASH/ASPC/NMA/PCNA Guideline for the Prevention, Detection, Evaluation, and Management of High Blood Pressure in Adults: A Report of the American College of Cardiology/American Heart Association Task Force on Clinical Practice Guidelines. Circulation. 2018 Oct 23;138(17):e484-e594.
[4] Halford JL, Wang LC, Choi SH, et al. Associations between alcohol intake and genetic predisposition with atrial fibrillation risk in a national biobank. Circ Genom Precis Med 2020. Dec;13(6):e003111
[5] Pathak RK, Middeldorp ME, Meredith M, et al. Long-Term Effect of Goal-Directed Weight Management in an Atrial Fibrillation Cohort: A Long-Term Follow-Up Study (LEGACY). *J Am Coll Cardiol.* 2015;65(20):2159-2169.
[6] Ye Y, Chen X, Han J, et al. Interactions between enhanced polygenic risk scores and lifestyle for cardiovascular disease, diabetes, and lipid levels. Circulation: Genomic and Precision Medicine. 2021;14:e003128

High Genetic Risk: Breast Cancer

Your patient's genetic analysis indicates that she is at HIGH GENETIC RISK (top 8th percentile) for breast cancer. This means that she has a greater than 2-fold higher risk of developing breast cancer compared to a woman with an average genetic risk score.

The polygenic score was calculated from hundreds of genetic markers in your patient's DNA. In prior studies, this score is highly correlated with the risk of breast cancer.[1] This genetic test does not account for other risk factors for breast cancer, such as family history, body-mass index, or smoking. It also does not account for genetic variants in the *BRCA1* or *BRCA2* genes.

Currently, there are no professional guidelines on how to manage a patient with high polygenic risk for breast cancer or whether that management should differ from the average population. This letter provides you with general recommendations for screening for and diagnosing breast cancer and helping your patients lower their risk for breast cancer.

Signs and Symptoms

Consider asking your patient about symptoms such as nipple discharge. On physical examination, look for breast masses or overlying skin changes.

Screening and Diagnosis

There are currently no professional guidelines on whether screening recommendations should differ for patients with high polygenic risk for breast cancer, compared with the average population. However, modeling studies suggest that women with a high-risk genetic breast cancer score might benefit from earlier screening (i.e. before age 50).[2]

Regardless of genetic risk score, it is important to make sure your patient's routine breast cancer screening is up-to-date. You and your patient should consider screening with mammography, as appropriate, based on age, family history, patient preferences, and prediction models such as the Gail model (https://bcrisktool.cancer.gov/) and others.[3,4]

Risk Factor Optimization

In addition to genetic predisposition for developing breast cancer, there are other factors that may raise or lower your patient's breast cancer risk, including smoking and alcohol consumption.[5,6,7]

Medications

It is not known whether selective estrogen receptor modulators like tamoxifen and raloxifene should be considered for women at high polygenic risk for breast cancer.

[1] Mavaddat N, Michailidou K, Dennis J, et al. Polygenic Risk Scores for Prediction of Breast Cancer and Breast Cancer Subtypes. *Am J Hum Genet.* 2019;104(1):21-34.
[2] van den Broek, J, Schechter C, van Ravesteyn, et al. Personalizing breast cancer screening based on polygenic risk and family history. *Journal of the National Cancer Institute*.2021;113(4):434-442
[3] Moyer VA. Risk assessment, genetic counseling, and genetic testing for BRCA-related cancer in women: U.S. Preventive Services Task Force recommendation statement. *Annals of Internal Medicine.* 2014;160(4):271-281.
[4] Gail MH, Brinton LA, Byar DP, et al. Projecting individualized probabilities of developing breast cancer for white females who are being examined annually. *Journal of the National Cancer Institute.*1989;81(24):1879-1886.
[5] Miller MD, Marty MA, Broadwin R, et al. The association between exposure to environmental tobacco smoke and breast cancer: a review by the California Environmental Protection Agency. *Prev Med.* 2007;44(2):93-106.
[6] Gram IT, Park SY, Kolonel LN, et al. Smoking and Risk of Breast Cancer in a Racially/Ethnically Diverse Population of Mainly Women Who Do Not Drink Alcohol: The MEC Study. *American Journal of Epidemiology.* 2015;182(11):917-925.
[7] White AJ, DeRoo LA, Weinberg CR, Sandler DP. Lifetime Alcohol Intake, Binge Drinking Behaviors, and Breast Cancer Risk. *American Journal of Epidemiology.* 2017;186(5):541-549.

High Genetic Risk: Colorectal Cancer

Your patient's genetic analysis indicates that he/she is at HIGH GENETIC RISK for colorectal cancer (top 5th percentile of genetic risk). This means that he/she has a greater than 2-fold higher risk of developing colorectal cancer compared to a person with an average genetic risk score.

The polygenic score was calculated from millions of genetic markers in your patient's DNA. In prior studies, this score was found to be highly correlated with risk for colorectal cancer (CRC).[1] This genetic test does not account for other disease risk factors, such as age and family history. It also does not account for genetic variants in the genes associated with hereditary nonpolyposis colorectal cancer (HNPCC) and familial adenomatous polyposis (FAP).

Currently, there are no professional guidelines on how to manage a patient with high polygenic risk for CRC or whether that management should differ from the average population. This letter provides you with general recommendations for screening for and diagnosing CRC and helping your patients lower their risk for CRC.

Signs and Symptoms

Consider asking your patient about symptoms such as unexplained weight loss, as well as any changes in bowel habits including bleeding, dark or maroon stools, constipation, irregular or inconsistent bowel movements.

Screening and Diagnosis

The US Preventive Services Task Force recommends that adults aged 45-75 be screened for CRC.[2] There are currently no professional guidelines on whether screening recommendations should differ for patients with high polygenic risk for CRC, compared with the average population.

However, it is important to make sure your patient's routine CRC screening is up-to-date. The VHA National Center for Health Promotion and Disease Prevention currently recommends the following screening strategies for patients at average risk[3]:

Screening Test	Test Frequency
Colonoscopy	Every 10 years
CT Colonography (virtual colonoscopy)	Every five years
Fecal Immunochemical Test (FIT)	Once a year
Fecal Occult Blood Test (FOBT)	Once a year
FIT-DNA Test	Once every one or three years
Flexible Sigmoidoscopy	Every five years; every 10 years with a FIT every year
Guaiac-based Fecal Occult Blood Test (gFOBT)	Once a year
Multi-target Stool DNA (mt-sDNA) Laboratory Test	Every three years

Risk Optimization

In addition to genetic predisposition for developing CRC, other factors that may increase your patient's risk, including smoking[4] and processed meat consumption[5]. If your patient smokes, consider referring him/her to a VA or other smoking cessation program. Observational studies suggest that individuals with high CRC genetic risk scores have lower risk if they have healthy values of BMI, physical activity, eat healthy amounts of fruits and vegetables, limit intake of alcohol and processed meats, and avoid smoking.[6]

[1] Huyghe JR, Bien SA, Harrison TA, et al. Discovery of common and rare genetic risk variants for colorectal cancer. *Nature Genetics.* 2019;51(1):76-87.
[2] https://www.uspreventiveservicestaskforce.org/uspstf/recommendation/colorectal-cancer-screening
[3] https://www.prevention.va.gov/index.asp
[4] Botteri E, Iodice S, Bagnardi V, Raimondi S, Lowenfels AB, Maisonneuve P. Smoking and colorectal cancer: a meta-analysis. *JAMA.* 2008;300(23):2765-2778.
[5] Bouvard V, Loomis D, Guyton KZ, et al. Carcinogenicity of consumption of red and processed meat. *The Lancet Oncology.* 2015;16(16):1599-1600.
[6] Choi et al. Am J Clin Nutr 2021. Healthy lifestyles, genetic modifiers, and colorectal cancer risk: a prospective cohort study in the UK Biobank. Am J Clin Nutr. 2021 Apr 6;113(4):810-820.

High Genetic Risk: Coronary Artery Disease

Your patient's genetic analysis indicates that he/she is at HIGH GENETIC RISK (top 10th percentile) for coronary artery disease (CAD). This means that he/she has a greater than 2-fold higher risk of developing CAD compared to a person with an average genetic risk score.

The genetic score was calculated from millions of genetic markers in your patient's DNA. In prior studies, this score was highly correlated with risk of CAD.[1] This genetic test does not account for other disease risk factors, such as age, alcohol intake, or other cardiovascular disease.

Currently, there are no professional guidelines on how to manage a patient with high genetic risk for CAD or whether that management should differ from the average population. This letter provides you with general recommendations for screening for and diagnosing CAD and helping your patients lower their risk for CAD.

Signs and Symptoms

Consider asking your patient about symptoms such as angina or dyspnea and consider diagnostic testing, if present.

Screening and Diagnosis

There are currently no professional guidelines on whether screening recommendations should differ for patients with high genetic risk for CAD, compared with the average population.

If CAD is suspected, consider screening with stress testing or coronary calcium computed tomography, particularly if the patient is starting a vigorous exercise program.

Risk Factor Optimization

In addition to genetic predisposition for developing CAD, there are other factors that may raise or lower your patient's CAD risk:

- Blood pressure: Patients should keep their blood pressure below 130/80[2]
- Cholesterol: Consider using lifestyle recommendation and statin therapy for cardiovascular disease prevention[3]
- Smoking and obesity: Refer patients to VA or other programs for smoking cessation and weight loss.[4]

Observational studies suggest that not smoking, maintaining a BMI<25 kg/m2, and meeting recommended physical activity levels might have even greater benefit in preventing CAD in patients with a high-risk genetic score.[5]

Observational data also suggest that individuals with intermediate (5-19.9%) 10-year ASCVD risk with a high-risk genetic score would benefit from starting a statin if not already on one.[4,5]

[1] Khera AV, Chaffin M, Aragam KG, et al. Genome-wide polygenic scores for common diseases identify individuals with risk equivalent to monogenic mutations. *Nature Genetics.* 2018;50(9):1219-1224.
[2] Whelton PK, Carey RM, Aronow WS, et al. 2017 ACC / AHA / AAPA / ABC / ACPM / AGS / APhA / ASH / ASPC / NMA / PCNA Guideline for the Prevention, Detection, Evaluation, and Management of High Blood Pressure in Adults: A Report of the American College of Cardiology/American Heart Association Task Force on Clinical Practice Guidelines. Circulation. 2018 Oct 23;138(17):e484-e594.
[3] Grundy SM et al. 2019. 2018 AHA / ACC / AACVPR / AAPA / ABC / ACPM / ADA / AGS / APhA / ASPC / NLA / PCNA Guideline on the Management of Blood Cholesterol: A Report of the American College of Cardiology/American Heart Association Task Force on Clinical Practice Guidelines. Circulation. 2019 Jun 18;139(25):e1082-e1143.
[4] Aragam KG, Dobbyn A, Judy R, et al. Limitations of Contemporary Guidelines for Managing Patients at High Genetic Risk of Coronary Artery Disease. J Am Coll Cardiol. 2020 Jun 9;75(22):2769-2780. doi: 10.1016/j.jacc.2020.04.027.
[5] Ye Y, Chen X, Han J, et al. Interactions between enhanced polygenic risk scores and lifestyle for cardiovascular disease, diabetes, and lipid levels. Circulation: Genomic and Precision Medicine. 2021;14:e003128

High Genetic Risk: Prostate Cancer

Your patient's genetic analysis indicates that he/she is at INCREASED GENETIC RISK (top 13th percentile) for prostate cancer. This means that he has a greater than 2-fold higher risk of developing prostate cancer compared to a man with an average genetic risk score.

The genetic score was calculated from millions of genetic markers in your patient's DNA. In prior studies, this score is highly correlated with risk for prostate cancer.[1] This genetic test does not account for other disease risk factors, such as age, family history, or race.[2]

Currently, there are no professional guidelines on how to manage a patient with high genetic risk for prostate cancer or whether that management should differ from the average population. This letter provides you with general recommendations for screening for and diagnosing prostate cancer and helping your patients lower their risk for prostate cancer.

Signs and Symptoms

Consider asking your patient about symptoms such as bone pain. On physical examination, look for prostate nodules or asymmetry and evaluate as appropriate.

Screening and Diagnosis

You and your patient should consider screening with prostate-specific antigen (PSA) testing as appropriate, based on age, family history, and patient preferences.

There are currently no professional guidelines on whether screening recommendations should differ for patients with high genetic risk for prostate cancer, compared with the average population. However, observational data suggest that PSA screening may have a more favorable benefit/risk ratio among individuals with higher genetic risk for prostate cancer.[3]

Risk Optimization

In addition to genetic predisposition for developing prostate cancer, other factors that may increase your patient's risk, including smoking.[4] If your patient smokes, consider referring him to a VA or other smoking cessation program.

Medications

5α-reductase inhibitors such as finasteride or dutasteride may reduce the incidence of prostate cancer risk among men at higher than average risk.[5] It is not known whether these medications reduce prostate cancer incidence among men with high polygenic risk for prostate cancer.

[1] Schumacher FR, Al Olama AA, Berndt SI, et al. Association analyses of more than 140,000 men identify 63 new prostate cancer susceptibility loci. *Nature Genetics.* 2018;50(7):928-936.
[2] Grossman DC, Curry SJ, Owens DK, et al. Screening for Prostate Cancer: US Preventive Services Task Force Recommendation Statement. *JAMA.* 2018;319(18):1901-1913.
[3] Callendar T, Emberton M, Morris S, et al. Polygenic risk-tailored screening for prostate cancer: A benefit-harm and cost-effectiveness study. PLoS Med 16(12): e1002998
[4] Islami F, Moreira DM, Boffetta P, Freedland SJ. A systematic review and meta-analysis of tobacco use and prostate cancer mortality and incidence in prospective cohort studies. *European Urology.* 2014;66(6):1054-1064.
[5] Prostate Cancer Prevention (PDQ®)-Health Professional Version. https://www.cancer.gov/types/prostate/hp/prostate-prevention-pdq

High Genetic Risk: Type 2 Diabetes

Your patient's genetic analysis indicates that he/she is at INCREASED GENETIC RISK (top 8th percentile) for type 2 diabetes mellitus. This means that he/she has a greater than 2-fold higher risk of developing type 2 diabetes compared to a person with an average genetic risk score.

The genetic score was calculated from millions of genetic markers in your patient's DNA. In prior studies, this score is highly correlated with risk for type 2 diabetes.[1] This genetic test does not account for other disease risk factors, such as body-mass index and sedentariness.

Currently, there are no professional guidelines on how to manage a patient with high genetic risk for type 2 diabetes or whether that management should differ from the average population. This letter provides you with general recommendations for screening for and diagnosing type 2 diabetes and helping your patients lower their risk for type 2 diabetes.

Signs and Symptoms

Consider asking your patient about symptoms such as polyuria, polydipsia, or neuropathy.

Screening and Diagnosis

There are currently no professional guidelines on whether screening recommendations should differ for patients with high genetic risk for type 2 diabetes, compared with the average population. However, it is important to make sure their routine type 2 diabetes screening is up-to-date.

You and your patient should consider screening with hemoglobin A1c, fasting glucose, or 2-hour oral glucose tolerance test, if appropriate.[2,3]

Risk Optimization

In addition to genetic predisposition for developing type 2 diabetes mellitus, other factors may increase your patient's risk:

- Overweight and obesity: If your patient is overweight or obese, consider referring them to the VA MOVE Program or other evidence-based weight loss program.[4]
- Diet: A Mediterranean-style diet high in fruits, vegetables, nuts, whole grains, and olive oil can lower the risk of type 2 diabetes.[5]
- Sedentariness: If appropriate, encourage your patient to engage in at least 150 minutes per week of moderate physical activity.[6]

Observational studies suggest that, compared with average-risk individuals, having healthy lifestyle factors (non-smoking, BMI<25 kg/m^2, exercise, and diet) is associated with even greater reduction in diabetes risk for patients with high-risk type 2 diabetes genetic scores.[7]

[1] Khera AV, Chaffin M, Aragam KG, et al. Genome-wide polygenic scores for common diseases identify individuals with risk equivalent to monogenic mutations. *Nature Genetics.* 2018;50(9):1219-1224.

[2] Classification and Diagnosis of Diabetes. *Diabetes Care.* 2017;40(Suppl 1):S11-s24.

[3] Siu AL. Screening for Abnormal Blood Glucose and Type 2 Diabetes Mellitus: U.S. Preventive Services Task Force Recommendation Statement. *Annals of Internal Medicine.* 2015;163(11):861-868.

[4] Knowler WC, Barrett-Connor E, Fowler SE, et al. Reduction in the incidence of type 2 diabetes with lifestyle intervention or metformin. *New England Journal of Medicine.* 2002;346(6):393-403.

[5] Salas-Salvado J, Bullo M, Babio N, et al. Reduction in the incidence of type 2 diabetes with the Mediterranean diet: results of the PREDIMED-Reus nutrition intervention randomized trial. *Diabetes Care.* 2011;34(1):14-19.

[6] Smith AD, Crippa A, Woodcock J, Brage S. Physical activity and incident type 2 diabetes mellitus: a systematic review and dose-response meta-analysis of prospective cohort studies. *Diabetologia.* 2016;59(12):2527-2545.

[7] Ye Y, Chen X, Han J, et al. Interactions between enhanced polygenic risk scores and lifestyle for cardiovascular disease, diabetes, and lipid levels. Circulation: Genomic and Precision Medicine. 2021;14:e003128
