# Supplementary material for: Development of a clinical polygenic risk score assay and reporting workflow
Source: Nat Med. 2022 Apr 18;28(5):1006–13. doi: 10.1038/s41591-022-01767-6 (PMC9117136; doi:10.1038/s41591-022-01767-6)
Supplement: Supplementary file 1 [file 41591_2022_1767_MOESM2_ESM.pdf]

nature portfolio

Corresponding author(s): Vassy

Last updated by author(s): Feb 2, 2022

Reporting Summary

Nature Portfolio wishes to improve the reproducibility of the work that we publish. This form provides structure for consistency and transparency in reporting. For further information on Nature Portfolio policies, see our Editorial Policies and the Editorial Policy Checklist.

Statistics

For all statistical analyses, confirm that the following items are present in the figure legend, table legend, main text, or Methods section.

n/a	Confirmed	
☐	☒	The exact sample size (*n*) for each experimental group/condition, given as a discrete number and unit of measurement
☐	☒	A statement on whether measurements were taken from distinct samples or whether the same sample was measured repeatedly
☒	☐	The statistical test(s) used AND whether they are one- or two-sided<br>*Only common tests should be described solely by name; describe more complex techniques in the Methods section.*
☐	☒	A description of all covariates tested
☒	☐	A description of any assumptions or corrections, such as tests of normality and adjustment for multiple comparisons
☒	☐	A full description of the statistical parameters including central tendency (e.g. means) or other basic estimates (e.g. regression coefficient) AND variation (e.g. standard deviation) or associated estimates of uncertainty (e.g. confidence intervals)
☐	☒	For null hypothesis testing, the test statistic (e.g. *F*, *t*, *r*) with confidence intervals, effect sizes, degrees of freedom and *P* value noted<br>*Give P values as exact values whenever suitable.*
☒	☐	For Bayesian analysis, information on the choice of priors and Markov chain Monte Carlo settings
☒	☐	For hierarchical and complex designs, identification of the appropriate level for tests and full reporting of outcomes
☐	☒	Estimates of effect sizes (e.g. Cohen's *d*, Pearson's *r*), indicating how they were calculated

Our web collection on statistics for biologists contains articles on many of the points above.

Software and code

Policy information about availability of computer code

Data collection	No software was used in the collection of data for this study.
Data analysis	Imputation of the MGBB data was performed using the Michigan Imputation Server (https://imputationserver.sph.umich.edu/) with ShapeIT (v2.r790) for phasing, Minimac3 (v2.0.1) for imputation, and 1000 Genome Project phase 3 data as the reference panel. PRS scores were calculated using PLINK (version 2.0a). PRS distributions were visualized using the density function in R (v4.0.3). R coefficients were calculated with RStudio (v1.1.383) with R (v4.0.3). Genotyping data filtering and gender information were conducted with bcftools v1.9. Imputation for the prospective assay was performed using EAGLE v2.4.1 for phasing, Minimac4 (v1.0.0) for imputation, and 1000 Genome Project phase 3 data as the reference panel.<br><br>Code used to adjust the PRS for population structure are available for download here: https://github.com/MGB-Personalized-Medicine/PRS-adjustment.

For manuscripts utilizing custom algorithms or software that are central to the research but not yet described in published literature, software must be made available to editors and reviewers. We strongly encourage code deposition in a community repository (e.g. GitHub). See the Nature Portfolio guidelines for submitting code & software for further information.

Data

Policy information about availability of data

All manuscripts must include a data availability statement. This statement should provide the following information, where applicable:

- Accession codes, unique identifiers, or web links for publicly available datasets
- A description of any restrictions on data availability
- For clinical datasets or third party data, please ensure that the statement adheres to our policy

The majority of the MGBB genotyped samples are deposited in dbGAP as part of the eMERGE consortium, Phase III (https://www.ncbi.nlm.nih.gov/projects/gap/cgi-bin/study.cgi?study_id=phs001584.v2.p2). Additional MGBB data were accessed under IRB protocol for this current study and are not publicly available due to restrictions on this the data. Data from the GenoVA Study trial will be made publicly available after study completion. The 1000 Genomes Project phase 3 dataset used in this study was v5a and was downloaded from ftp://ftp.1000genomes.ebi.ac.uk/vol1/ftp/release/20130502/. PRS summary statistics were obtained from the Polygenic Score (PGS Catalog; https://www.pgscatalog.org/; PGP ID = PGP000006, PGS ID = PGS000016; PGP ID = PGP000006, PGS ID = PGS000013; PGP ID = PGP000006, PGS ID = PGS000014; and PGS ID = PGS000007, PGP ID = PGP000002) and Cancer-PRSWeb (https://prsweb.sph.umich.edu/; PRSWEB_PHECODE153_CRC-Huyghe_PT_MGI_20191112, PRS tuning parameter: 3.98107170553497e-07; and PRSWEB_PHECODE185_Pca-PRACTICAL_LASSOSUM_MGI_20191112, PRS tuning parameter: s0.5_Lambda0.00695192796177561).

Field-specific reporting

Please select the one below that is the best fit for your research. If you are not sure, read the appropriate sections before making your selection.

☒ Life sciences ☐ Behavioural & social sciences ☐ Ecological, evolutionary & environmental sciences

For a reference copy of the document with all sections, see nature.com/documents/nr-reporting-summary-flat.pdf

Life sciences study design

All studies must disclose on these points even when the disclosure is negative.

Sample size	PRS were developed and validated in all 36,423 Mass General Brigham Biobank participants with available genotype data. We show that this sample size was sufficient to replicated the known PRS-disease phenotype associations.
Data exclusions	No data were excluded from the analysis.
Replication	MGBB, clinical, and GenoVA Study samples were analyzed once on each platform described. NIST reference samples were analyzed in replicates.
Randomization	Samples were not allocated into experimental groups. Models used to develop the PRS adjusted for population structure included the first 4 ancestry-informative principal components.
Blinding	Analysts were blinded to the case/control status of biobank participants when calculating each PRS.

Reporting for specific materials, systems and methods

We require information from authors about some types of materials, experimental systems and methods used in many studies. Here, indicate whether each material, system or method listed is relevant to your study. If you are not sure if a list item applies to your research, read the appropriate section before selecting a response.

Materials & experimental systems

n/a	Involved in the study	
☒	☐	Antibodies
☒	☐	Eukaryotic cell lines
☒	☐	Palaeontology and archaeology
☒	☐	Animals and other organisms
☐	☒	Human research participants
☐	☒	Clinical data
☒	☐	Dual use research of concern

Methods

n/a	Involved in the study	
☒	☐	ChIP-seq
☒	☐	Flow cytometry
☒	☐	MRI-based neuroimaging

Human research participants

Policy information about studies involving human research participants

Population characteristics	Among 36,423 MGBB participants, mean (SD) age was 58.8 (17.1) years (range 9-106), 19,719 (54.1%) were female, and 5706

Population characteristics	(15.7%) were of reported race other than white [30,716 (84.3%) white, 1,807 (5.0%) Black, 786 (2.2%) Asian, and 3,113 (8.5%) of other/unknown race]. Among the first 227 GenoVA Study enrollees aged 50-70, 119 (52%) are of non-white reported race/ethnicity, and 78 (34%) currently identify as women.
Recruitment	All patients of affiliated hospitals and clinics are eligible to participate in the MGB Biobank. VA Boston patients aged 50-70 without known diagnoses of the 6 targets diseases are eligible for enrollment. Both the MGB Biobank and GenoVA Study populations have the potential for healthy volunteer bias. The samples used for genome sequencing derive from 22 clinical samples of patients who have undergone clinical sequencing, but these results should not meaningfully bias the comparison of sequence and genotype data for PRS calculation.
Ethics oversight	Analyses of the genomic and MGB Biobank samples and data has been reviewed and approved by the Mass General Brigham IRB (2019P001933). Analyses for the prospective pipeline, including the use of prior clinical samples, was conducted under the Mass General Brigham IRB (2004P001056); all individuals with clinical testing, including those with genome sequencing data, gave consent for clinical genomic sequencing and all individual data were de-identified. The GenoVA Study is approved by the VA Boston Healthcare System (#3241) and Harvard Medical School IRB (IRB19-0594), and all enrollees provided written informed consent.

Note that full information on the approval of the study protocol must also be provided in the manuscript.

Clinical data

Policy information about clinical studies

All manuscripts should comply with the ICMJE guidelines for publication of clinical research and a completed CONSORT checklist must be included with all submissions.

Clinical trial registration	The GenoVA Study: ClinicalTrials.gov Identifier: NCT04331535
Study protocol	https://clinicaltrials.gov/ct2/show/NCT04331535
Data collection	GenoVA Study participants were recruited from the VA Boston Healthcare System (see https://clinicaltrials.gov/ct2/show/NCT04331535) beginning on July 17, 2020. Recruitment is ongoing. Data were collected from medical records, participant report, and from genotype array data from a blood or saliva specimen.
Outcomes	The primary and secondary outcomes are predefined on clinicaltrials.gov. The primary outcome of the study will be assessed at the end of the trial and will be time-to-diagnosis both of undiagnosed prevalent cases of the 6 target conditions and incident cases during the study period, as adjudicated by expert clinical blinded chart review. Secondary outcomes will be diagnostic testing (from medical records and patient report), patient activation (using the Patient Activation Measure), healthcare costs (from administrative data and microcosting analysis), and medication adherence (Voils Medication Adherence Survey).
